# Publisher Correction: Bookend: precise transcript reconstruction with end-guided assembly

**DOI:** 10.1186/s13059-022-02725-8

**Published:** 2022-07-13

**Authors:** Michael A. Schon, Stefan Lutzmayer, Falko Hofmann, Michael D. Nodine

**Affiliations:** 1 Cluster of Plant Developmental Biology, Laboratory of Molecular Biology, Wageningen University & Research, Wageningen, 6708, PB The Netherlands; 2 Gregor Mendel Institute (GMI), Austrian Academy of Sciences, Vienna Biocenter (VBC), Dr. Bohr-Gasse 3, 1030 Vienna, Austria

**Correction: Genome Biol 23, 143 (2022)**

**https://doi.org/10.1186/s13059-022-02700-3**

Following publication of the original article [1], the authors noticed that Figs. 4 and 5 were transposed during production. Below is the correct layout of Figs. 4 and 5. The original article [1] has been corrected. The publishers apologise for the error.

**Fig. 4:**
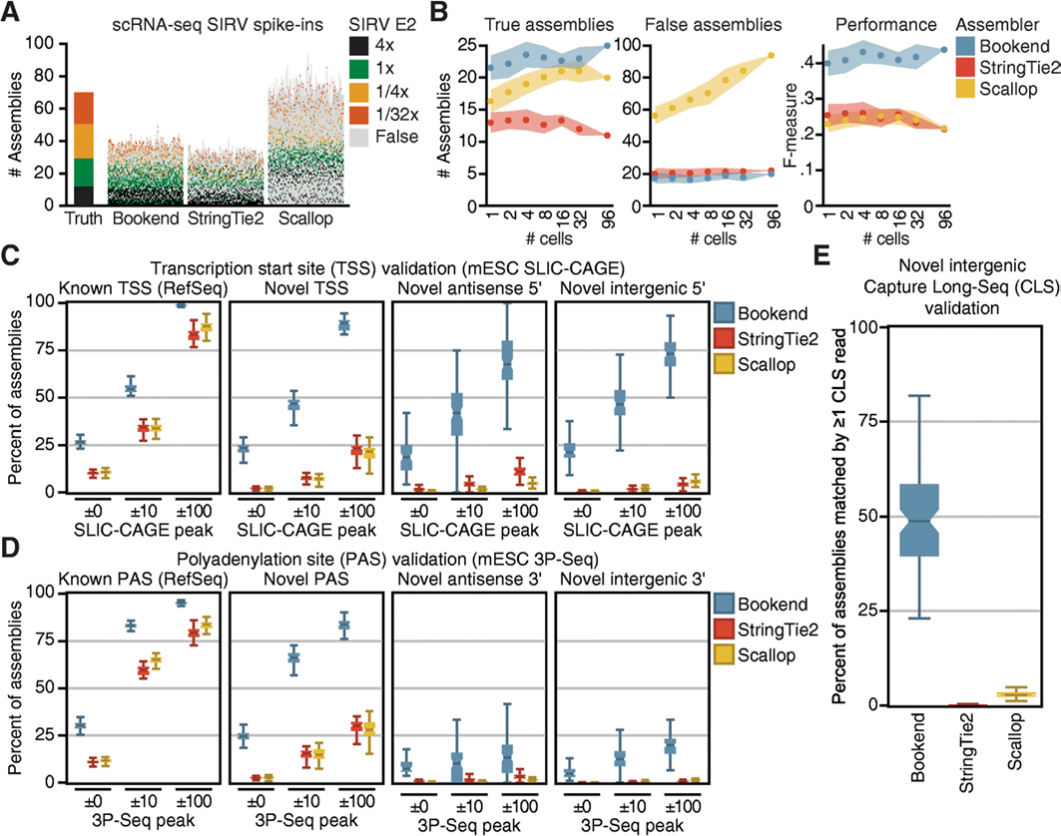
Bookend performance on single mouse cells. **A** Reconstruction of Spike-In RNA Variants (SIRVs) from 96 paired-end 100 bp SMARTer libraries of single mESCs. Each vertical bar depicts the assemblies from one cell, ordered from highest (bottom) to lowest (top) estimated abundance. Colored boxes match a true isoform of the given input concentration; gray boxes are false assemblies. **B** SIRV assembly performance as a function of increasing sequencing depth. F-measure (right) is the harmonic mean of sensitivity and precision. **C** Boxplots showing percent validation of 5′ ends with SLIC-CAGE support within the given windows for 96 single mESC assemblies. **D** Boxplots as in (**C)** showing 3′ end validation by 3P-Seq peaks. **E** Percent of intergenic assemblies (no overlap with RefSeq) in single cells which have ≥1 matching Capture Long-Seq read from the mouse CLS atlas

**Fig. 5:**
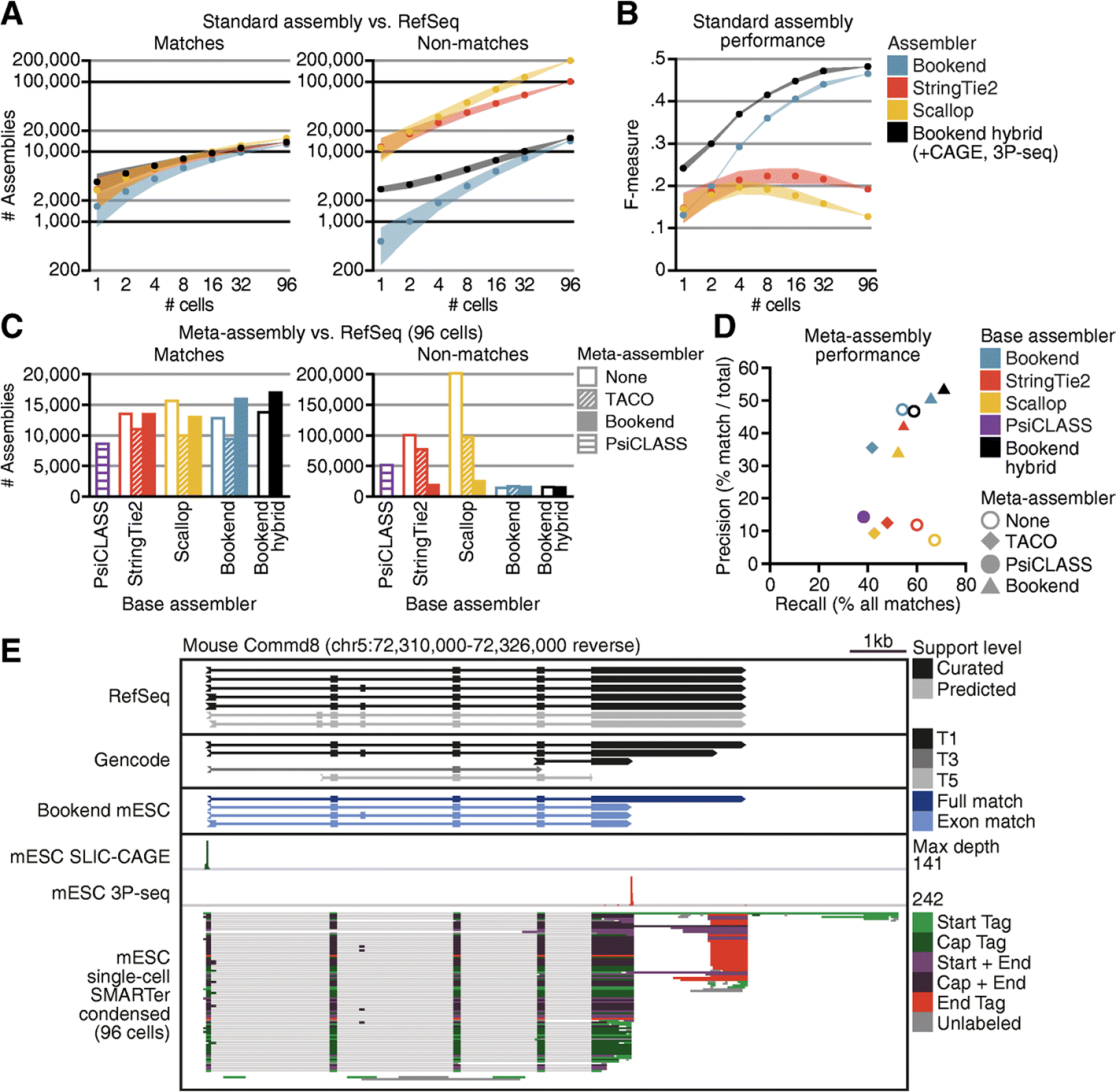
End-guided meta-assembly accurately integrates single-cell data. **A** Performance of assemblers with input from increasing numbers of single mESC cells. Assemblies with a matching exon chain to a RefSeq transcript (left) or no match to a RefSeq transcript (right). **B** F-measure of assemblies, where recall is the proportion of all transcripts assembled by ≥1 strategy and precision is matches/total assemblies. **C** Comparison of Bookend meta-assembly to standard assembly and other meta-assemblers. Number of RefSeq-matching transcripts assembled (left) or the number of non-matches (right). **D** Precision/recall plot of the 12 assemblies from **C**; recall and precision calculated as in (**B)**. **E** IGV browser image of the Commd8 gene. From top to bottom: RefSeq, Gencode, and Bookend mESC annotations, 5′ ends from mESC SLIC-CAGE, 3′ ends from mESC 3P-seq, Bookend-condensed partial assemblies from 96 single mESCs
