# Supplementary material for: Bookend: precise transcript reconstruction with end-guided assembly
Source: Genome Biol. 2022 Jun 29;23:143. doi: 10.1186/s13059-022-02700-3 (PMC9245221; doi:10.1186/s13059-022-02700-3)
Supplement: Supplementary file 1 — Additional file 1: Figure S1. The Bookend workflow. Figure S2. Nucleotide-level precision of Arabidopsis assembly 5′ and 3′ ends. Figure S3. Artifacts in long-read data. Figure S4. Single mESC assembly details. Figure S5. Meta-assembly details. Table S1. Floral bud Smart-seq2 end-labeled read mapping statistics. Table S2. Long-read validation of floral bud assemblies by class. Table S3. Floral bud hybrid assembly details. Table S4. End-labeling and alignment of single mESCs. Table S5. GffCompare performance statistics for mESC meta-assemblies. Supporting notes. Detailed information about End Labeled Read file format and assembly algorithms. [file 13059_2022_2700_MOESM1_ESM.pdf]

SUPPLEMENTAL MATERIAL

Additional file 1 of
Bookend: precise transcript reconstruction with end-guided assembly

This document serves as an Appendix to [Schon et al. 2022], and it contains descriptions of algorithms used by the end-guided transcript assembler Bookend. Source code can be found at https://github.com/Gregor-Mendel-Institute/bookend.

The package can be installed from the Python Package Index on any system with Python 3.6+ using the command `pip install bookend-rna`.

CONTENTS

SUPPLEMENTAL FIGURES **2**
Figure S1. The Bookend workflow . . . 2
Figure S2. Nucleotide-level precision of Arabidopsis assembly 5′ and 3′ ends . . . 3
Figure S3. Artifacts in long-read data . . . 4
Figure S4. Single mESC assembly details . . . 5
Figure S5. Meta-assembly details . . . 6

SUPPLEMENTAL TABLES **7**
Table S1. Floral bud Smart-seq2 end-labeled read mapping statistics . . . 7
Table S2. Long-read validation of floral bud assemblies by class . . . 8
Table S3. Floral bud hybrid assembly details . . . 9
Table S4. End-labeling and alignment of single mESCs . . . 10
Table S5. GffCompare performance statistics for mESC meta-assemblies . . . 12

SUPPORTING NOTES **13**
The End Labeled Read file format . . . 13
Assembly Algorithms . . . 14
Generate Chunks . . . 15
Tag Clustering . . . 16
Calculate Membership Matrix . . . 18
Calculate Overlap Matrix . . . 19
Collapse Linear Chains . . . 21
Generate Overlap Graph . . . 23
Resolve Containment . . . 24
Greedy Paths . . . 25
Assign Weights to Paths . . . 27
Path Filtering . . . 28

Additional file 2 contains all Supplemental Datasets as tabs in an Excel spreadsheet:

Dataset 1.	Bookend Floral Bud, hybrid assembly Arabidopsis stage 12 inflorescence
Dataset 2.	Classification of Bookend Floral Bud transcripts against TAIR10 and Araport11
Dataset 3.	Transcription start site (TSS) usage in five floral organs for known and novel TSSs
Dataset 4.	Bookend mESC, hybrid assembly of mouse embryonic stem cells
Dataset 5.	Classification of Bookend mESC transcripts against RefSeq and Gencode

Additional file 3 is the Bookend user guide, which describes all utilities and their arguments. An up-to-date User Guide is maintained on the GitHub repository.

SUPPLEMENTAL FIGURES

Figure S1. The Bookend workflow

A Workflow diagram showing the computational steps to generate an end-guided assembly from raw FASTQ RNA-seq files with Bookend. **B** Schematic of the Membership Matrix produced from a hypothetical collection of reads from various sequencing methods; blue- frag is included, pink- frag is excluded. Elements that include or exclude every frag are considered complete, e.g. the first and last long reads.

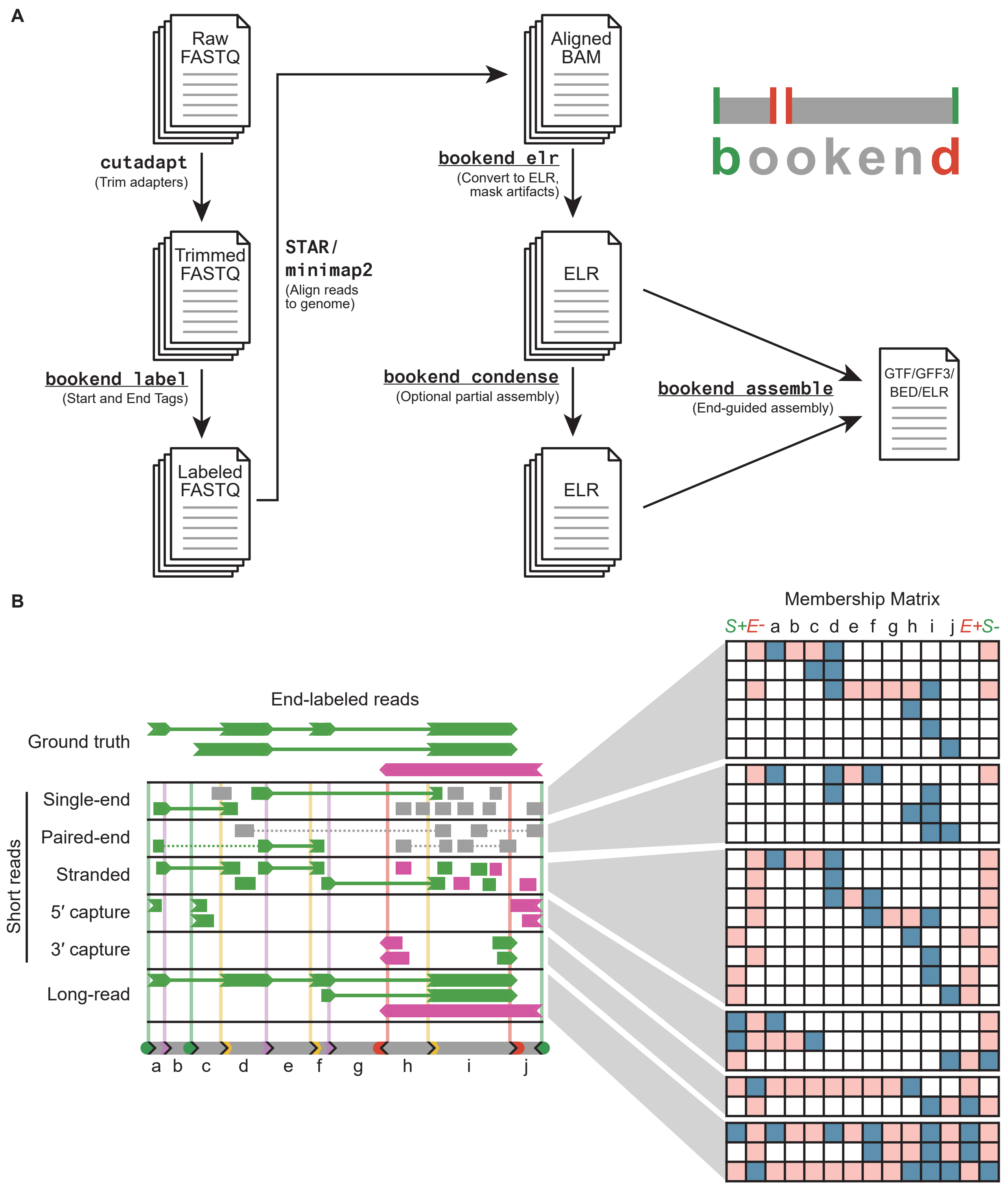

Figure S2. Nucleotide-level precision of Arabidopsis assembly 5′ and 3′ ends

A Percent of the unique set of 5′ ends from different assemblies of floral bud RNA, or from reference annotations (TAIR10, Araport11) overlapping assembled loci, that fall on or adjacent to the peak position of a CAGE cluster from Thieffry et al. 2020. **B** Validation as in **A** for the set of unique 3′ ends against Direct RNA-Seq (DRS) data from Sherstnev et al. 2012. **C** (Top) Nucleotide fold enrichment over background frequencies in a ±50nt window around the peak positions of all CAGE clusters; (bottom left) nucleotide enrichment around assembly 5′ ends ≤ 100nt from a TAIR10 TSS; (bottom right) enrichment around all other assembly 5′ ends. **D** Enrichments displayed as in **C** for DRS clusters and assembly 3′ ends. **E** Estimated read coverage distribution for assembled transcripts grouped by their classification against TAIR10. Center line- median coverage. Outliers are not shown.

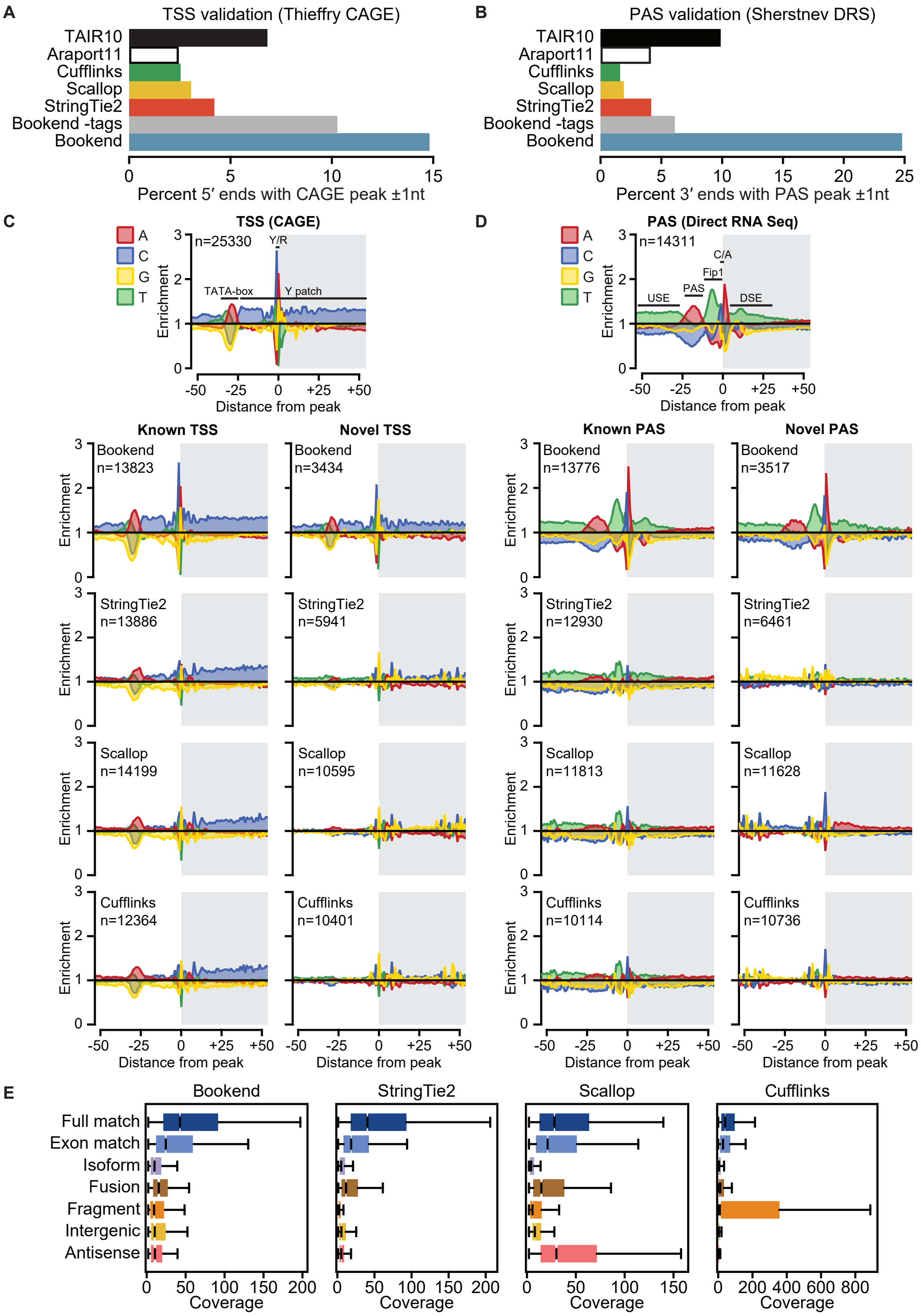

Figure S3. Artifacts in long-read data

IGV browser image of the Arabidopsis *MIA* locus. From top to bottom: PacBio FLNC reads, colored by 3′ label type; Bars demarcating genomic regions of 10 or more consecutive A's (10 or more T's on the reverse strand); Direct RNA Seq (DRS) 3′ end abundance; nanoPARE 5′ end capped and noncapped read abundance; Smart-seq2 coverage depth; TAIR10 reference; Assemblies colored by class vs. TAIR10. **B** (Upper panel) Nucleotide frequency enrichment in a ±50bp window around transcription start sites (TSS) identified by CAGE (Thieffry et al. 2020). (Lower panel) Nucleotide enrichment around 5′ ends of transcripts constructed from PacBio reads by Iso-seq3 (top), StringTie2 (middle), and Bookend (bottom) at sites overlapping a TAIR10 TSS (left), novel TSS at a known gene (middle), and novel antisense or intergenic loci (right). **C** Precision/recall plot of assemblers on Smart-seq2 (short reads) and/or Pacbio (long reads).

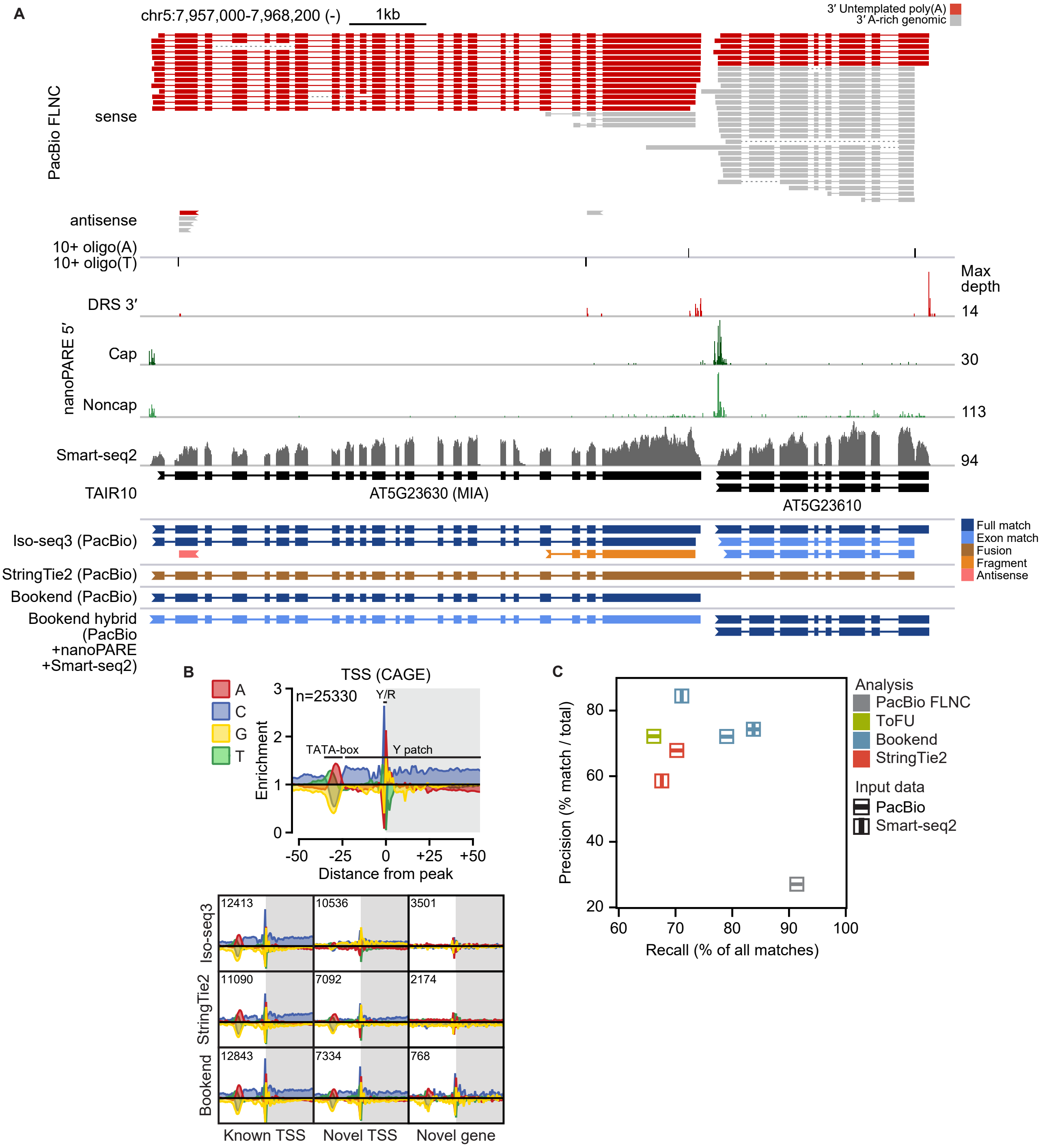

Figure S4. Single mESC assembly details

A IGV browser image of the synthetic SIRV6 locus. (Top) Mix E2 spike-in concentrations for 18 distinct RNA molecules. (Bottom) Assembled isoforms of pooled data from Natarajan et al. 2019 of 96 SMARTer libraries of single mESCs with SIRV spike-ins. Transcripts are colored by the concentration of their matching SIRV transcript, if it exists. **B** Box plots of processing time (left) and peak memory usage (right) to assemble 96 mESCs with five transcript assemblers. **C** Box plots of recall and precision comparing four assembly methods to Bookend with and without filtering incomplete transcripts. **D** Precision/recall plots for assemblies of 96 mESCs whose RNA was split to generate three RNA-seq libraries from two different sequencing protocols. Black points incorporate mESC SLIC-CAGE data from Cvetesic et al. 2018 and mESC 3P-Seq data from Nam et al. 2013 in a hybrid assembly.

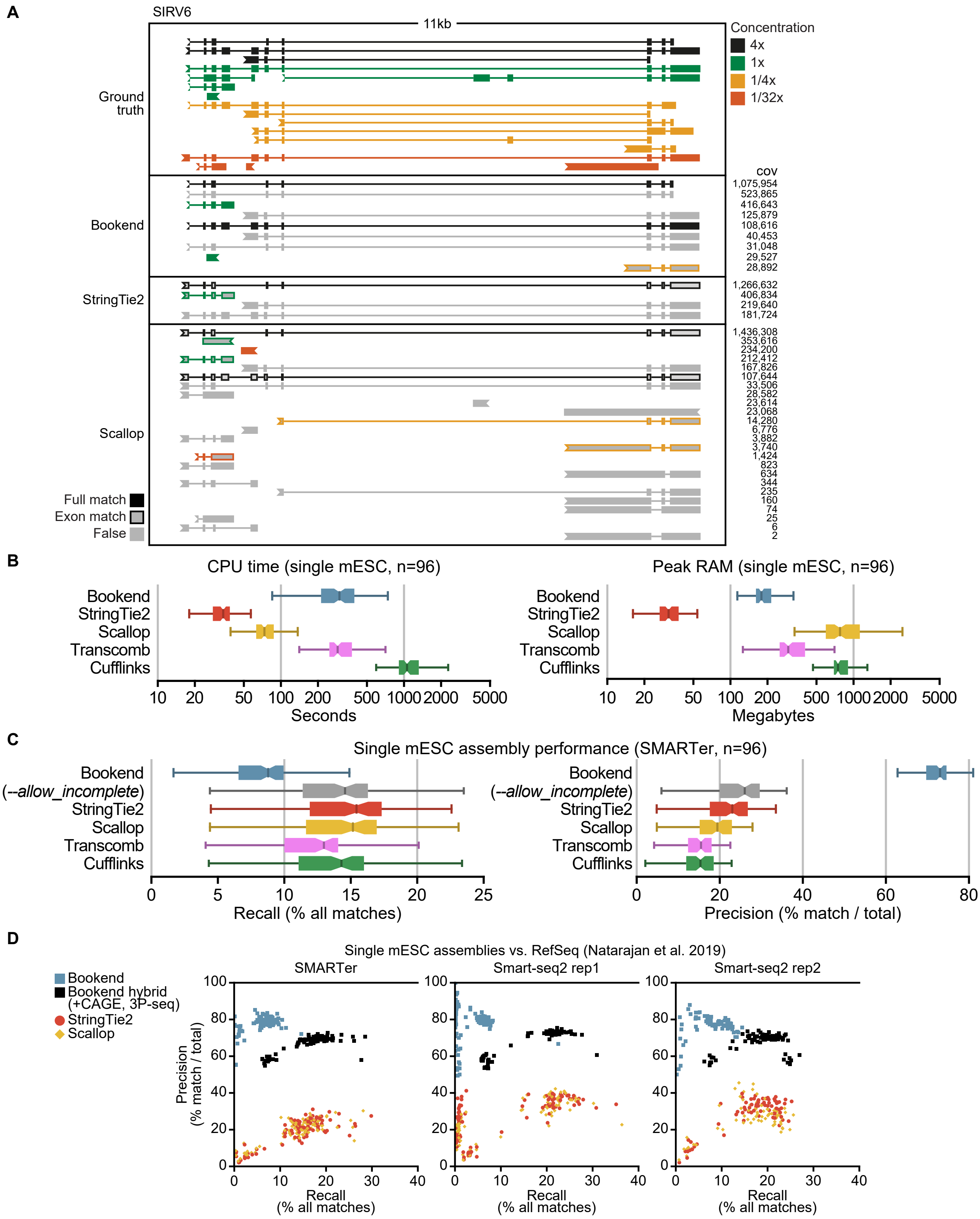

Figure S5. Meta-assembly details

A IGV browser image of the mouse Gga1 gene. Tracks from top to bottom: read abundance of mESC SLIC-CAGE and 3P-seq; Bookend partial assembly of SMARTer data from 96 mESC cells; meta-assembly by Bookend, PsiClass, and TACO. **B** Classification of all assembled transcripts with and without meta-assembly. **C** Change in transcript abundance by class after meta-assembly by TACO or Bookend. **D** Euler diagram of the number of shared exon chains between Bookend mESC, RefSeq and Gencode annotations at loci assembled by Bookend.

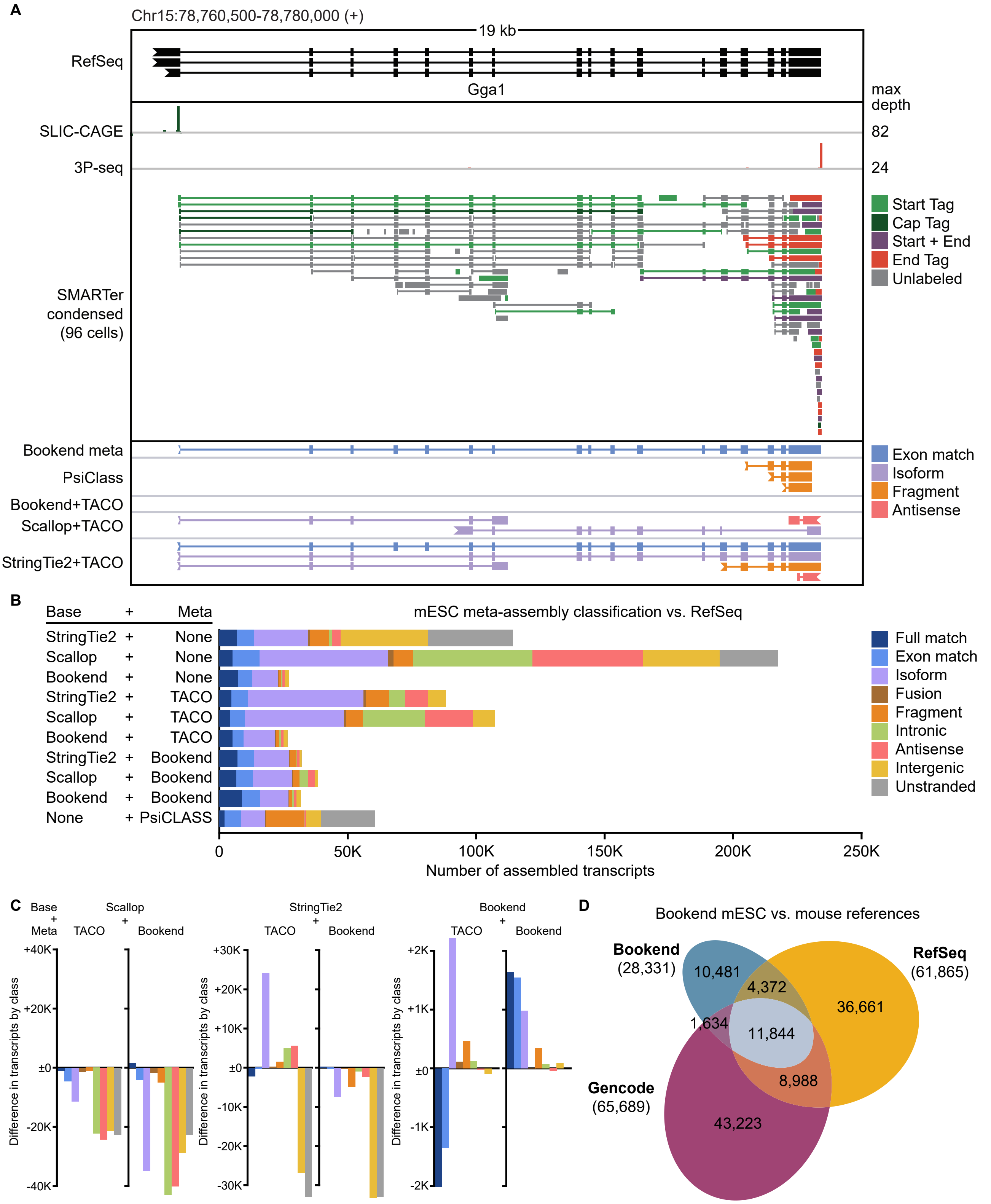

SUPPLEMENTAL TABLES

Table S1. Floral bud Smart-seq2 end-labeled read mapping statistics

End-labeled reads identified in three Smart-seq2 replicates from 5ng of Arabidopsis floral bud total RNA, sequenced single-end 50bp mode on an Illumina Hi-Seq 2500.

LABEL

Read type	Rep 1	Rep 2	Rep 3	Merged
Raw reads	26,714,548	24,104,566	24,626,064	75,445,178
5′ Label	743,292	948,127	657,093	2,348,512
3′ Label	194,038	389,972	211,447	795,457

ALIGN

Read type	Rep 1	Rep 2	Rep 3	Merged
Unique mappers	20,218,104 (76%)	16,552,901 (69%)	18,655,520 (76%)	55,426,525 (73%)
Start Tag	446,529 (60%)	523,009 (55%)	416,070 (63%)	1,385,608 (59%)
End Tag	126,315 (65%)	252,673 (65%)	149,980 (71%)	528,968 (66%)
Multi-mappers	5,266,353 (20%)	5,609,624 (23%)	4,645,067 (19%)	15,521,044 (21%)
Start Tag	213,413 (29%)	273,929 (29%)	165,207 (25%)	652,549 (28%)
End Tag	31,979 (16%)	53,612 (14%)	23,970 (11%)	109,561 (14%)

FILTER

Read type	Rep 1	Rep 2	Rep 3	Merged
5′ TSO artifacts	71,921 (11%)	108,081 (14%)	74,085 (13%)	254,087 (12%)
5′ uuG Caps	51,748 (8%)	48,398 (6%)	39,601 (7%)	139,747 (7%)
3′ poly(A) artifacts	52,047 (33%)	91,573 (30%)	50,166 (29%)	193,786 (30%)

FINAL

Read type	Rep 1	Rep 2	Rep 3	Merged
Unlabeled	24,666,221	21,059,302	22,545,360	68,270,883
Start Tags	536,273	640,459	467,591	1,644,323
Cap Tags	51,748	48,398	39,601	139,747
End Tags	126,315	214,712	123,784	464,811

Table S2. Long-read validation of floral bud assemblies by class

Breakdown of assembled transcripts by their classification against the closest TAIR10 reference transcript, and the number of transcripts in each class that have at least one full match (exon chain and ±100 ends) in PacBio long-read sequencing data from 10µg of *Arabidopsis thaliana* stage 12 floral bud total RNA.

CLASSIFICATION

Class	Bookend	Bookend -tags	StringTie2	Scallop	Cufflinks
Full match	**12019**	7809	10674	9068	5480
Exon match	4139	5851	4579	**6717**	5571
Isoform	1915	2409	5337	8317	**9728**
Fusion	185	1101	1049	1439	**3737**
Fragment	362	1325	1813	3431	**3955**
Intergenic	399	279	530	1113	**1160**
Antisense	112	70	145	**3049**	341
Intronic	6	3	12	**51**	13
Ambiguous	0	819	1910	**50185**	6025
Known	**16158**	13660	15253	15785	11051
Novel	2979	5187	8886	17400	**18934**
Total	19137	18847	24139	**33185**	29985

VALIDATION

Class	Bookend	Bookend (-tags)	StringTie2	Scallop	Cufflinks
Full match	**11239 (94%)**	7070 (91%)	9671 (91%)	7925 (87%)	4619 (84%)
Exon match	**3033 (73%)**	2450 (42%)	2124 (46%)	1961 (29%)	1252 (23%)
Isoform	**903 (47%)**	660 (27%)	1216 (23%)	1630 (20%)	693 (7%)
Fusion	**56 (30%)**	42 (4%)	60 (6%)	87 (6%)	36 (1%)
Fragment	**116 (32%)**	63 (8%)	80 (4%)	83 (2%)	65 (2%)
Intergenic	**147 (37%)**	21 (8%)	46 (9%)	68 (6%)	20 (2%)
Antisense	**38 (34%)**	5 (7%)	7 (5%)	150 (5%)	9 (3%)
Intronic	**1 (17%)**	0 (0%)	0 (0%)	2 (4%)	0 (0%)
Ambiguous	N/A	N/A	N/A	N/A	N/A
Known	**14272 (88%)**	9520 (70%)	11795 (77%)	9886 (63%)	5871 (53%)
Novel	**1261 (42%)**	791 (15%)	1409 (16%)	2020 (12%)	823 (4%)
Total	**15533 (81%)**	10311 (55%)	13204 (55%)	11906 (36%)	6694 (22%)

Table S3. Floral bud hybrid assembly details

Classification against the closest match in TAIR10 or Araport11 of transcripts assembled from a set of complementary datasets from floral bud RNA. Short reads- Smart-seq2; Start reads- nanoPARE; Long reads- PacBio.

Bookend arguments:
(Long reads only) *–min_proportion .01 –min_len 60 –max_gap 50 –min_cov 1 –cap_bonus 2*
(Hybrid) *–min_proportion .01 –min_len 60 –max_gap 50 –min_cov 1.5 –cap_bonus 2 –cap_filter 0.1*
StringTie2 arguments:
(Long reads only) *-f .01 -m 60 -c 1 -M 1 -L*
(Hybrid) *-f .01 -m 60 -c 1.5 -M 1 -L -g 50 -s 1.5 –mix*

LONG READS ONLY

Class	PacBio FLNC	(unique)	Iso-seq3	(unique)	ToFU	StringTie2	Bookend
Full match	292394	(16630)	23071	(12882)	14479	13131	**15140**
Exon match	158554	(8104)	13122	(3490)	**5333**	4209	4886
Isoform	33754	(20546)	3279	(2747)	**3856**	3018	3475
Fusion	5303	(1341)	502	(206)	596	**838**	403
Fragment	46952	(19525)	7045	**(4804)**	781	609	399
Intergenic	25346	(8177)	2457	(1784)	**2305**	1628	282
Antisense	7865	(4441)	956	(828)	**1542**	548	438
Intronic	401	(101)	42	**(20)**	16	8	10
Ambiguous	0	(0)	0	(0)	0	**22**	0
Known	450948	(24734)	36193	(16372)	19812	17340	**20026**
Novel	119621	(54131)	14281	**(10389)**	9096	6671	5007
Total	570569	(78865)	50474	(26761)	**28908**	24011	25033
(% Known)	79.0%	(31.4%)	71.7%	(61.2%)	68.5%	72.2%	**80.0%**

HYBRID ASSEMBLY

Assembler	StringTie2	Bookend	Bookend	Bookend	Bookend	
Class	Long+Short	Long+Short	Long+Start	Short+Start	Long+Short+Start	(require cap)
Full match	14718	16626	14978	13921	16341	**(16743)**
Exon match	5468	5753	5209	5577	**6312**	(5814)
Isoform	4645	5382	5688	2664	**6047**	(5703)
Fusion	**1026**	587	462	328	583	(495)
Fragment	**1395**	313	432	670	504	(514)
Intergenic	1827	425	231	484	**577**	(389)
Antisense	414	476	323	408	**637**	(550)
Intronic	12	**10**	3	5	6	(11)
Ambiguous	**877**	0	0	0	0	(0)
Known	20186	22379	20187	19498	**22653**	(22557)
Novel	10196	7193	7139	4559	**8354**	(7662)
Total	30382	29572	27326	24057	31007	(30219)
(% Known)	66.4%	75.7%	73.9%	81.0%	73.1%	(74.6%)

Table S4. End-labeling and alignment of single mESCs

Mapping statistics for 96 single mESC SMARTer libraries from Natarajan et al. 2019.

Cell	Unlabeled	5′ label	3′ label	Aligned	Start Tag	Cap Tag	(%)	End Tag	(%)
1	9608668	439193	100121	8934461	258330	79340	0.9%	37906	0.4%
2	9831074	618059	118804	9607068	365454	96924	1.0%	54055	0.6%
3	12146979	641146	127258	11425200	386469	105849	0.9%	51691	0.5%
4	6335181	298745	79348	5750133	178094	51310	0.9%	31671	0.6%
5	7514264	427582	90844	7177000	251555	73783	1.0%	40753	0.6%
6	6192857	424987	71214	5568044	238054	64005	1.1%	29469	0.5%
7	9413990	557275	105944	8676900	308285	97282	1.1%	41137	0.5%
8	7146811	478394	109538	6626337	303099	55831	0.8%	41087	0.6%
9	12209527	656470	119208	11653781	364251	126662	1.1%	51548	0.4%
10	4943634	249114	57765	4150020	133363	44176	1.1%	22076	0.5%
11	10289384	579612	156501	9540995	344615	107138	1.1%	67970	0.7%
12	6296290	354539	36954	5744670	207542	57459	1.0%	15386	0.3%
13	11062529	608742	107064	10588553	402291	90084	0.9%	38529	0.4%
14	5859896	372904	57864	5597543	221374	58681	1.0%	25575	0.5%
15	7438431	377316	85287	6600211	216701	60159	0.9%	31166	0.5%
16	9318662	489053	77267	8804817	287566	85378	1.0%	29135	0.3%
17	9654938	591123	111696	8821047	345010	95892	1.1%	46811	0.5%
18	4176594	309959	77647	3386566	196545	14120	0.4%	21449	0.6%
19	10166487	646012	87377	9584997	400128	99395	1.0%	34685	0.4%
20	4513269	374249	70382	3592280	232724	13498	0.4%	17983	0.5%
21	5911031	367655	77492	5715353	230948	68676	1.2%	34725	0.6%
22	8533714	500707	100164	8057641	293859	101787	1.3%	44715	0.6%
23	5507278	446779	71288	4598916	301960	17407	0.4%	19768	0.4%
24	7007715	436446	81182	6404774	243859	75132	1.2%	33581	0.5%
25	3995373	323171	74361	3632465	237703	10268	0.3%	20365	0.6%
26	5412175	277064	69361	5014494	165316	45054	0.9%	25527	0.5%
27	4498372	268910	56498	4148402	168253	40980	1.0%	23122	0.6%
28	29923526	1235689	302270	29149150	791759	206991	0.7%	117971	0.4%
29	10165433	565658	123935	9849721	358781	93532	0.9%	48662	0.5%
30	6088334	309792	72381	5791867	198551	44964	0.8%	24476	0.4%
31	7560915	419014	82924	6738642	265942	45899	0.7%	25400	0.4%
32	5485800	345511	78723	4856333	199379	50828	1.0%	29350	0.6%
33	5373713	373940	66041	4823649	224204	52800	1.1%	29194	0.6%
34	6485920	426953	71948	5608916	225477	65596	1.2%	29468	0.5%
35	6632255	360820	74635	5934034	219050	49897	0.8%	26091	0.4%
36	5977435	506299	90559	5824146	368469	27634	0.5%	32330	0.6%
37	2705467	189226	58195	1931832	118339	4561	0.2%	28556	1.5%
38	6035540	371889	79129	5753194	233286	64046	1.1%	36886	0.6%
39	4425629	292098	59420	4081161	168990	48537	1.2%	26558	0.7%
40	6388799	427916	66878	6408595	301594	60211	0.9%	32197	0.5%
41	7985364	460244	115172	7245203	269110	82244	1.1%	48552	0.7%
42	7125365	342965	73899	6819032	226665	48831	0.7%	23736	0.3%
43	3268434	287751	64027	1932186	146951	5797	0.3%	25172	1.3%
44	6776069	348272	70412	5796199	198263	51087	0.9%	26426	0.5%
45	6267559	334111	94890	5700986	202717	57249	1.0%	38813	0.7%
46	7858871	514789	85073	7257444	324611	81400	1.1%	35532	0.5%
47	6456318	295115	47795	5819580	171799	50144	0.9%	17653	0.3%
48	6404736	411525	74035	5627612	232133	63004	1.1%	29733	0.5%

Table S4, continued.

Cell	Unlabeled	5′ label	3′ label	Aligned	Start Tag	Cap Tag	(%)	End Tag	(%)
49	3389101	340785	115033	3422875	284973	8238	0.2%	33683	1.0%
50	5667716	363316	70312	4977135	222168	45158	0.9%	24698	0.5%
51	2690373	227903	58683	1946712	141653	5730	0.3%	29509	1.5%
52	6928921	362190	107121	6545706	225017	60246	0.9%	40119	0.6%
53	9835754	726933	136436	9463908	428107	107183	1.1%	62678	0.7%
54	3011944	270978	65486	2543453	185334	13107	0.5%	29921	1.2%
55	5142534	484427	92112	5059348	385759	18370	0.4%	36733	0.7%
56	7140952	395151	86458	6594524	251851	60822	0.9%	34039	0.5%
57	6466893	413749	63792	5598598	217828	69633	1.2%	29085	0.5%
58	3300888	266911	71045	2541725	166731	8887	0.3%	18473	0.7%
59	5043344	309051	46246	4926209	192103	48126	1.0%	20633	0.4%
60	6986624	481048	80193	6427797	279353	77828	1.2%	36246	0.6%
61	3351952	234887	76558	3319998	176381	26927	0.8%	41700	1.3%
62	3396729	339973	85217	3274103	264194	12512	0.4%	33795	1.0%
63	6461789	421328	66538	5632766	236839	57459	1.0%	26779	0.5%
64	6404975	422427	85385	5785467	270948	41527	0.7%	26209	0.5%
65	6557014	342169	117742	5717291	193178	52509	0.9%	42129	0.7%
66	3335945	307264	73995	2564465	184833	11539	0.4%	22667	0.9%
67	6375049	322527	59493	5944082	199964	47648	0.8%	22887	0.4%
68	8177038	404930	93646	7564092	252086	70170	0.9%	34101	0.5%
69	5485331	301692	84083	4864549	167140	47594	1.0%	32817	0.7%
70	14155483	792368	132139	13194440	467058	123415	0.9%	55436	0.4%
71	7022871	439562	91457	6477060	283655	61701	1.0%	33721	0.5%
72	7114718	466219	72834	6382720	262582	74644	1.2%	31258	0.5%
73	7494258	359929	110643	7266395	224032	63724	0.9%	45323	0.6%
74	9200518	599103	116324	8846834	377761	89071	1.0%	49789	0.6%
75	4351846	413743	90042	4214629	315500	19554	0.5%	33950	0.8%
76	4062440	346688	83752	3356938	233841	12302	0.4%	24880	0.7%
77	6264611	353118	100568	5758030	210097	62049	1.1%	43787	0.8%
78	6425634	332547	63487	5703753	202498	45756	0.8%	23191	0.4%
79	7234869	381668	113175	6540410	221080	65323	1.0%	48511	0.7%
80	10168955	356816	144538	8923837	176870	53756	0.6%	51863	0.6%
81	6613647	359383	55306	6380549	217473	63718	1.0%	26495	0.4%
82	3280612	308671	87547	2792536	216501	8298	0.3%	23508	0.8%
83	6292413	380969	59590	5645459	212510	67444	1.2%	25334	0.4%
84	5619685	508203	75195	5003815	349041	31826	0.6%	25397	0.5%
85	9277709	604069	92078	9040233	420256	79640	0.9%	41817	0.5%
86	6115076	342837	118423	5611657	200182	62551	1.1%	48331	0.9%
87	8466897	479130	137519	8090541	289579	83847	1.0%	56166	0.7%
88	5910776	308345	101811	5749104	198101	53210	0.9%	41257	0.7%
89	8795051	480325	131394	7941499	273086	88094	1.1%	49065	0.6%
90	6935880	397900	130562	6470810	230171	70897	1.1%	54555	0.8%
91	8114433	474650	129503	7215724	267064	79081	1.1%	45991	0.6%
92	3884626	214058	72777	3214197	115207	34069	1.1%	27498	0.9%
93	8932179	552463	99937	8764917	340984	98729	1.1%	44467	0.5%
94	8641039	533914	100584	7795662	283006	100413	1.3%	42540	0.5%
95	6281335	391868	78347	5496105	206090	62725	1.1%	29948	0.5%
96	5000892	288069	78217	4875119	174688	53544	1.1%	34999	0.7%

Table S5. GffCompare performance statistics for mESC meta-assemblies

Comparison of mESC assemblies with and without meta-assembly vs. overlapping RefSeq transcripts. Command-line argument: `gffcompare -R --strict-match --no-merge -e 100 -s GRCm39.fasta -r [RefSeq] [Assembly]`

SENSITIVITY

Base assembler	Meta-assembler	Nucleotide	Exon	Intron	Intron chain	Transcript
PsiCLASS	PsiCLASS	41.6	44.0	49.0	13.0	13.1
StringTie2	None	50.3	55.1	59.5	15.4	15.4
StringTie2	TACO	43.3	53.7	58.4	13.3	13.1
StringTie2	Bookend	49.6	63.8	66.2	21.3	21.1
Scallop	None	**58.9**	54.1	60.5	17.0	16.9
Scallop	TACO	45.7	53.4	57.6	11.6	11.5
Scallop	Bookend	49.5	62.1	64.5	20.8	20.7
Bookend	None	58.1	67.4	70.0	20.0	20.1
Bookend	TACO	46.3	57.9	59.4	18.0	18.0
Bookend	Bookend	56.6	**67.8**	**70.4**	**24.7**	**24.7**
Bookend+	None	57.4	67.1	69.7	19.8	19.9
Bookend+	Bookend	56.7	68.7	71.5	25.0	25.0
Bookend+	Bookend (stringent)	57.9	70.2	73.0	25.5	25.5

+ Hybrid assembly with mESC CAGE and 3P-seq data

PRECISION

Base assembler	Meta-assembler	Nucleotide	Exon	Intron	Intron chain	Transcript
PsiCLASS	PsiCLASS	36.7	61.0	**95.8**	28.0	14.6
StringTie2	None	32.3	52.0	86.2	29.6	12.1
StringTie2	TACO	38.8	53.0	78.8	15.9	12.1
StringTie2	Bookend	**77.3**	79.9	92.3	38.6	38.4
Scallop	None	16.1	35.4	82.2	21.5	6.8
Scallop	TACO	28.6	48.0	83.1	18.2	8.6
Scallop	Bookend	59.6	74.6	92.5	38.1	30.5
Bookend	None	56.8	84.1	95.0	50.8	44.7
Bookend	TACO	67.2	79.3	90.4	40.8	35.4
Bookend	Bookend	63.6	**84.5**	94.0	**52.1**	**46.5**
Bookend+	None	58.1	83.9	95.1	52.3	44.5
Bookend+	Bookend	66.6	85.8	94.5	56.2	49.9
Bookend+	Bookend (stringent)	77.8	87.8	94.8	58.3	54.1

+ Hybrid assembly with mESC CAGE and 3P-seq data

SUPPORTING NOTES

The End Labeled Read file format

End Labeled Read (ELR) files are defined in two parts: a header that builds an index of reference chromosomes (#C) and read sources (#S), and a 7-column body with the following contents:

1. Chromosome index [int]
2. Alignment start position [int]
3. Alignment length (including gaps) [int]
4. Strand {+, -, .}
5. ELCIGAR string describing alignment labels and gaps [str]
6. Source of read [int]
7. Weight of all reads matching this description [float]

ELCIGAR strings describe the position and labels of all aligned segments of a read, patterned off of the BAM/SAM format CIGAR strings but with additional End Label information. They are strings of Character/Number pairs with one trailing character ($[CN]_xC$), where C is a label and N is a numeric distance on the genome. Each label annotates the end of the associated span as one of the following:

S	Start Tag (RNA 5′ end)
C	Capped Tag (5′ end with untemplated G)
E	End Tag (RNA 3′ end)
D	Splice junction donor
A	Splice junction acceptor
.	Unspecified gap/end

For example, the ELCIGAR for a 50bp paired-end read of a 185nt cDNA fragment with no introns or end labels would be ".50.85.50.". A full-length transcript with the ELCIGAR "$S256D800A128D800A512E$" has 3 exons of 256, 128, and 512nt, respectively, and 2 introns that are both 800nt.

Short indels and mismatches are not recorded in ELCIGAR strings, but the number of alignment errors within each exonic region (between adjacent SD, CD, AD, and AE pairs) is tallied. If this tally exceeds the user-specified error rate as a proportion of the number of aligned bases in that exon, then it is removed from the ELCIGAR and the surrounding exons are bridged with an unspecified gap (.. pair). This setting prevents the use of splice junctions from especially error-prone alignments, which can be common in some long-read sequencing protocols.

During assembly, the "weight" column will be used to determine read coverage depth for exonic frags and for end positions. However, if a Start Tag, Capped Tag, or End Tag is lowercase (s, c, e), `bookend assemble` will treat these ends as having a weight of 1 instead of the read weight. This is beneficial for partial assembly of sparsely-labeled samples.

Assembly Algorithms

This section lays out pseudocode for the essential elements of end-guided assembly. Pseudocode is written in a "Pythonic" way, e.g. indices and ranges are 0-indexed open.

NOTE: Some basic mathematical notation is used.

$\sum$	Sum
$\|A\|$	Number of elements in A
$a : b$	Iterate from a to b
$a\|b$	a or b
$a \in A$	a is in set A
$A \bigcup B$	Union of sets A and B
$A \subset B$	A is a subset of B
$A \setminus B$	The subset of A not shared with B

First, the input reads must be grouped into Chunks. To assemble each chunk, the branchpoints must first be generated, a list of all start clusters, end clusters, splice donors, and splice acceptors on each strand. Splice junctions are cataloged first and rare junctions are removed. Then, Tag Clustering is performed to generate two strand-specific lists of Start Clusters and Tag Clusters. Each adjacent pair of branchpoints demarcates a "frag", and the Membership of *reads* is calculated in which each read either includes (1), excludes (-1) or does not overlap with (0) each frag. Rows with identical memberships are combined to produce the Membership Matrix, and from here the Overlap Matrix can be calculated. The Overlap Matrix is further simplified by collapsing Linear Chains, after which the Overlap Graph can be defined. The weights of shorter reads fully contained within one or more longer reads are redistributed via the Resolve Containment algorithm.

Assembly is carried out on the Overlap Graph using the Greedy Paths algorithm, where a set of optimal paths through the Overlap Graph is produced. After addition of each path, the weights of all reads are assigned proportionally to each path. Finally, the optimal set of paths is filtered to remove incomplete and low-confidence models, and the remaining paths are output as assembled transcripts.

The assembly environment is built around the *RNAseqMapping* object, which acts as a general container for RNA-seq data or transcript models, and possesses a number of methods for conversion from/to various RNA-seq file formats (BED, BED12, BAM/SAM, ELR, GTF/GFF3). The data contained in each *RNAseqMapping* object includes:

RNAseqMapping attributes:

name	data type	description
chrom	*int*	Chromosome number
source	*int*	Source number
strand	$\{1, -1, 0\}$	Alignment strand (forward, reverse, unstranded)
ranges	*list*	Ordered list of (left,right) aligned segments
splice	*list*	List of bools $\|ranges\| - 1$; is the gap an intron?
weight	*float*	Abundance (counts) of the Object
s_tag	*bool*	Object contains a Start Tag?
capped	*bool*	Object contains a Capped Tag?
e_tag	*bool*	Object contains an End Tag?
complete	*bool*	Object is a full-length gapless transcript
is_reference	*bool*	Object came from a reference annotation file
condensed	*bool*	Object is a product of `bookend condense`
s_len	*int*	Length of trimmed Start Tag
e_len	*int*	Length of trimmed End Tag
attributes	*dict*	Container for additional $key : value$ information

Generate Chunks

Assembly begins by building coherent chunks of overlapping reads that could putatively be considered ”genes” or ”loci”. Each time there is a gap greater than the specified `--maxgap` between the rightmost edge of the reads in the chunk and the left edge of the next read, a chunk is completed. Within this chunk, false positive splice junctions may skip over gaps. To filter spurious spliced reads, read coverage (sum of read.weight) is calculated for each base in the chunk, and the weight of each splice junction is compared to the coverage of its flanking positions to discard all reads with sufficiently rare junctions.

Algorithm 1: GenerateChunks

Data: *Reads* = a sorted list of *RNAseqMapping* objects
g = the maximum tolerable gap length
p = the minimum proportion of coverage needed to treat a splice junction as valid
Result: An ordered set of *Chunks*, each a subset of *Reads* separated by a gap $> g$

```
begin
    chunk ← An empty list
    chrom ← −1
    for read ∈ Reads do
        if chunk is empty then
            spanRight ← read.right
        else if read.chrom ≠ chrom or read.left > spanRight + g then
            junctions ← Unique set of spliced positions in chunk
            junctionWeights ← Dict of ∑ r.weight for r ∈ chunk which contain each j ∈ junctions
            cov ← ∑ r.weight for all r ∈ chunk overlapping each genomic position in chunk
            badJunctions ← All j ∈ junctions where
              junctionWeights[j] < p ∗ max(cov[j.left], cov[j.right])
            chunk ← Subset of r ∈ chunk that do not contain badJunctions
            breaks ← Runs > g where no reads in chunk overlap
            for b ∈ breaks do
                subchunk ← Subset of r ∈ chunk where r.right < b
                yield subchunk
            chunk ← [read] /* Start a new chunk containing only the current read */
            spanRight ← read.right
        else
            spanRight ← max(spanRight, read.right)
        chunk.append(read)
    yield chunk
```

Tag Clustering

From an array of Start Tag or End Tag positions (the 5′ or 3′ terminus of all tagged reads, respectively), Tag Clusters are determined independently on each strand. Clusters are not allowed to span same-stranded splice junctions, which could yield malformed transcript models with termini (defined as the peak signal position within a Tag Cluster) internal to a splice site. For the same reason, same-stranded Start Clusters and End Clusters that overlap are further subdivided in a way that prevents malformed transcripts with a 3′ end upstream of the 5′ end. These two rules are implemented through the algorithms **GenerateTagClusters** and **ResolveOverlap**, respectively. See the diagrams below for examples of malformed transcript models:

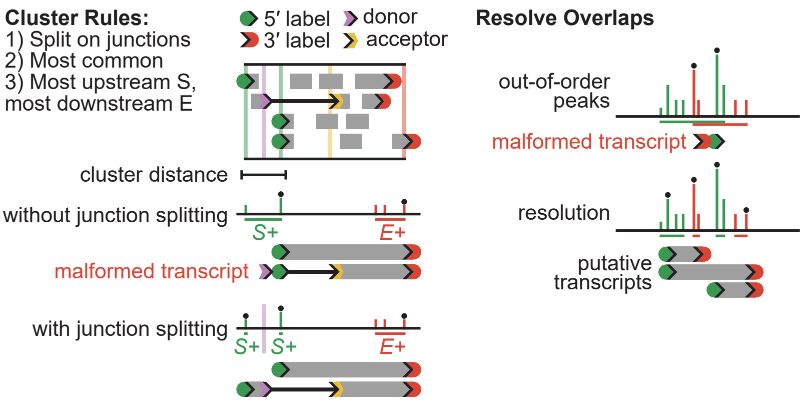

Algorithm 2: GenerateTagClusters

Data: *counts* = Array of tag weights of one type (Start, End) and one strand (+,-) by genomic position
coverage = Array of weights of all overlapping reads by genomic position
junctions = Array of splice donor and acceptor site positions on the same strand as *counts*
strandRatio = Array by genomic position of inferred proportion of overlapping reads that align to the input strand [0-1]
overhang = Minimum distance allowed between a splice site and a Tag Cluster
g = the maximum tolerable gap between tags of the same cluster
p = minimum proportion of tag weights to use as signal threshold
Result: List of *TagCluster* objects that record left, right, and most abundant positions

```
begin
    values ← counts² * strandRatio/coverage where counts ≠ 0
    prohibited ← iterator({junctions ± overhang, −1})
    threshold ← p * Σ values
    passPositions ← positions where values > threshold
    TagClusters ← An empty list
    boundary ← −1
    cluster ← TagCluster(passPositions[0])
    while prohibited exists and boundary < cluster.left do
        boundary ← next(prohibited)
    for p ∈ order(passPositions[1 :], key = values[passPositions[1 :]]) do
        crossedBoundary ← boundary > −1 and p > boundary
        if crossedBoundary or p − g > cluster.right then
            TagClusters.append(cluster)
            cluster ← TagCluster(p)
            if crossedBoundary then
                while prohibited exists and boundary < p do
                    boundary ← next(prohibited)
        else
            cluster.add(p)
    TagClusters.append(cluster)
    return TagClusters
```

Algorithm 3: ResolveOverlap

```
Data: LeftRanges = Sorted list of TagCluster objects of type S+ or E−
RightRanges = Sorted list of TagCluster objects complementary to the type of LeftRanges (E+ if
S+, S− if E−)
Result: A copy of LeftRanges and RightRanges in which overlapping TagClusters with out-of-order
        peaks have been split.
begin
    NewLefts, NewRights ← Empty lists
    AddedLefts, AddedRights ← Empty sets
    for L ∈ LeftRanges do
        for R ∈ RightRanges do
            if R.peak not in AddedRights then
                if R.right < L.left then
                    NewRights.append(R)
                    AddedRights.add(R.peak)
                    continue
                else if R.left > L.right then
                    if L.peak not in AddedLefts then
                        NewLefts.append(L)
                        AddedLefts.add(L.peak)
                    continue
                if L.right > R.left and L.left < R.right then
                    if L.peak > R.peak then
                        LeftPositions ← subset of L.positions < R.peak
                        if |LeftPositions| > 0 then
                            SplitLL ← TagCluster(LeftPositions)
                            NewLefts.append(SplitLL)
                        RightPositions ← subset of L.positions > R.peak
                        if |RightPositions| > 0 then
                            SplitLR ← TagCluster(RightPositions)
                            NewLefts.append(SplitLR)
                            if R.right > SplitLR.peak then
                                LeftPositions ← subset of R.positions < SplitLR.peak
                                RightPositions ← subset of R.positions > SplitLR.peak
                                if |LeftPositions| > 0 then
                                    NewRights.append(TagCluster(LeftPositions))
                                if |RightPositions| > 0 then
                                    NewRights.append(TagCluster(RightPositions))
                            else
                                NewRights.append(R)
                                AddedRights.add(R.peak)
                        AddedLefts.add(L.peak)
                        AddedRights.add(R.peak)
                    else if L.peak not in AddedLefts then
                        NewLefts.append(L)
                        AddedLefts.add(L.peak)
                if L.peak not in AddedLefts then
                    NewLefts.append(L)
                    AddedLefts.add(L.peak)
                for R ∈ RightRanges do
                    if R.peak not in AddedRights then
                        NewRights.append(R)
                        AddedRights.add(R.peak)
    return NewLefts, NewRights
```

Calculate Membership Matrix

Using the set of splice junctions and tag clusters a locus can be divided into $frags$, defined as a $(left, right)$ range of positions between two adjacent "branchpoints": splice donor/acceptor sites, the upstream-most position of a Start Tag Cluster, or the downstream-most position of an End Tag Cluster. The "membership" of a $read$ is an integer array of length $|frags| + 4$ that records whether it overlaps (1), does not overlap (0), or excludes (-1) the $frag$. 4 extra integers record presence/absence/exclusion of the 4 rag types: $S+$, $E+$, $S-$, $E-$. A $frag$ excluded by a $read$ cannot be part of the same transcript; either the $read$ passes over $frag$ via an intron, or the $read$ has a Start/End Tag that terminates outside $frag$. *Reads* with the same membership arrays are identical for the purposes of assembly and are collapsed into a single *element*. The Membership Matrix records the membership arrays of all *elements* in a matrix of shape $|elements| \times |frags|$. The weight of each element is calculated as the total number of bases in reads contained in the *element* divided by the number of nucleotides included in the *element frags*. *Element* weights are recorded separately in a Weight Matrix of shape $|elements| \times |sources|$.

Algorithm 4: CalculateMembershipMatrix

Data: $Reads$ = a sorted list of $RNAseqMapping$ objects
$frags$ = Ordered list of $(left, right)$ positions of all adjacent non-overlapping branchpoints
$TagClusters$ = Collection of clusters defined by **GenerateTagClusters**
Result: $Membership$: A $|elements| \times |frags| + 4$ matrix associating each $frag$ and Tag type to each unique $element$
$Weights$: A $|elements| \times |sources|$ float matrix recording summed weights by source of each $element$

```
begin
    Sp, Ep, Sm, Em ← |frags|, |frags| + 1, |frags| + 2, |frags| + 3
    elements ← An empty set
    elementWeights ← An empty dict
    for i ∈ |Reads| do
        read ← Reads[i]
        membership ← [0] × (|frags| + 4)
        if read.strand = 1 then
            membership[Sp] ← int(read.s_tag and read starts in a Start+ Cluster)
            membership[Ep] ← int(read.e_tag and read ends in an End+ Cluster)
            membership[Sm] ← −1
            membership[Em] ← −1
        else if read.strand = −1 then
            membership[Sm] ← int(read.s_tag and read starts in a Start− Cluster)
            membership[Em] ← int(read.e_tag and read ends in an End− Cluster)
            membership[Sp] ← −1
            membership[Ep] ← −1
        previousFrag ← −1
        for j ∈ |read.ranges| do
            left, right ← read.ranges[j]
            leftFrag ← which frag contains left
            rightFrag ← which frag contains right
            membership[leftFrag : (rightFrag + 1)] ← 1
            if j > 0 then
                gapRange ← (prevFrag + 1) : leftFrag
                membership[gapRange] ←
                    { −1,  if read.splice[j − 1]
                    { 1,   if !read.splice[j − 1] and gapRange overlaps no junctions
                    { 0,   otherwise
        hash ← asString(membership)
        elements.add(hash)
        elementWeights[hash, read.source] ←
          read.weight ∗ Σ_{r∈read.ranges}(r[1] − r[0]) / Σ_{m∈members}(frags[m][1] − frags[m][0])
    orderedElements ← sort(asArray(h) for h ∈ elements)
    Membership ← Matrix(orderedElements)
    Weights ← Matrix(elementWeights[e] for e ∈ orderedElements)
    return Membership, Weights
```

Calculate Overlap Matrix

The Overlap Matrix (O) is a $|elements| \times |elements|$ square matrix that describes the relationship between each *element* pair. It is asymmetric; each pair of *elements* {a,b} has two coordinates on the matrix, O_{ab} and O_{ba}. There are four possible overlap relationships: "does not overlap" (0), "extends" (1), "cannot extend" (-1), and "is subset of" (2). For example, if *element* b contains all members of a, then "a is subset of b" and $O_{ab} = 2$. All *elements* necessarily are subsets of themselves, so the main diagonal of O is 2. "Extension" can be understood as the ability to traverse (from left to right) the $frags$ of one *element* onto the $frags$ of another. Examples of *element* pairs are given in the table below. The symbol set $\{-,\ ,+\}$ is used for *membership* values of $\{-1, 0, 1\}$, respectively. Because alignments can contain gaps, it is possible that "a extends b" and "b extends a". Positive values in O will form edges in a directed graph (the **Overlap Graph**). Negative values in O can form an undirected graph (incompatibility is symmetric) that imposes limits on traversal of the Overlap Graph.

Examples of Overlap between *element* pairs

	Membership	O_{ab}	O_{ba}
a<br>b	`++`<br>`  ++`	0	0
a<br>b	`++`<br>` ++`	1	0
a<br>b	`+++`<br>` ++`	0	2
a<br>b	`++-++`<br>` +++`	-1	-1
a<br>b	`++ ++`<br>` +++`	1	1

Algorithm 5: GetOverlap

Data: a = A single row of the Membership Matrix (integer array length $|frags| + 4$)
b = A second row of the Membership Matrix
Result: (O_{ab}, O_{ba}) : Overlap relationship of $a \to b$ and $b \to a$, respectively
begin
  $info_a \leftarrow \sum a \neq 0$
  $info_b \leftarrow \sum b \neq 0$
  $buffer \leftarrow (False, False, False, False)$ /* Stores *membership* transition states for $(a_i, a_{i-1}, b_i, b_{i-1})$ */
  $shared, ext_{ab}, ext_{ba} \leftarrow 0$
  $overlapping, in_a, in_b \leftarrow False$
  for $i \in 0 : |a|$ **do**
    if $(a_i = 1$ *and* $b_i = -1)$ *or* $(a_i = -1$ *and* $b_i = 1)$ **then**
      return $(-1, -1)$
    $shared.add(int(a_i = b_i$ and $a_i \neq 0))$
    $overlapping \leftarrow overlapping$ or $(a_i + b_i = 2)$
    $buffer \leftarrow (a_i \neq 0, buffer[0], b_i \neq 0, buffer[2])$
    $ext_{ab}.add(int(in_a$ and $buffer = (False, True, True, True)))$
    $ext_{ba}.add(int(in_b$ and $buffer = (True, True, False, True)))$
    $in_a \leftarrow ia \neq 0$ and $(in_a$ or $a_i = 1)$
    $in_b \leftarrow ib \neq 0$ and $(in_b$ or $b_i = 1)$
  if $shared \leq 0$ **then**
    return $(0, 0)$
  if $shared = info_a$ **then**
    $O_{ab} \leftarrow 2$
  else if $shared = info_b$ **then**
    $O_{ba} \leftarrow 2$
  else
    $O_{ab} \leftarrow int(overlapping$ and $ext_{ab} > 0)$
    $O_{ba} \leftarrow int(overlapping$ and $ext_{ba} > 0)$
  return (O_{ab}, O_{ba})

Algorithm 6: CalculateOverlapMatrix

Data: $Membership$ = Output from **CalculateMembershipMatrix**
$Strand$ = Array of length $|elements|$ of alignment strands $\{-1, 0, 1\}$
Result: $Overlap$: Overlap Matrix, integer array of shape $|elements| \times |elements|$

```
begin
    Overlap ← 0-matrix of shape |elements| × |elements|
    for a ∈ 0 : |elements| do
        for b ∈ a : |elements| do
            if b = a then
                Overlap_ab, Overlap_ba ← 2
            else
                if (Strand_a = 1 and Strand_b = −1) or (Strand_a = −1 and Strand_b = 1) then
                    Overlap_ab, Overlap_ba ← −1
                    next
            Overlap_ab, Overlap_ba ← GetOverlap(a,b)
    return Overlap
```

Collapse Linear Chains

When considering paths that join sets of *elements*, some relationships between *elements* are trivial, meaning that there are no branches in the path. For assembly, a non-branching set of *elements* can be treated as a single *element* that contains the sum of *membership* information in the set. To identify all non-branching *element* sets (Linear Chains), a strategy was developed to traverse the positive edges of the Overlap Matrix via Depth First Search (DFS). The *elements* are ordered by increasing information content, and all *elements* are visited by DFS. Upon postvisit, a "chain number" is assigned to the *element* based on the set of chains seen during the visit. The strategy defined below is sufficient to resolve both trivial cases and nested cases, where gapped reads create cycles in the Overlap Matrix. After labeling each *element* according to its *chain*, a new Reduced Membership Matrix (RMM) and Reduced Overlap Matrix (ROM) can be created from the set of unique *chains*, rather than all unique *elements*. The example below shows the $Membership$ and *Overlap* of a Locus before (left) and after (right) reduction. This Locus contains (1) a long linear chain, (2) a nested loop, and (3) a "long read" *element* that has complete information. The *chain* of each *element* defined by **IdentifyLinearChains** is shown in the rightmost column. The RMM and ROM produced by **CollapseLinearChains** are shown to the right, where *elements* that gained information from their *chain* are capitalized. It is important to note here that even though every *element* except h are contained in a, they are assigned to a different *chain* than a because, for example, *elements* b through g could belong to a valid path containing either h or i, but a is only compatible with i. If the contained *elements* were merged into a chain with a, then no complete path containing h could exist. For *Overlap* and ROM, the symbol set $\{-, \ , \circ, \bullet\}$ is used to replace $\{-1, 0, 1, 2\}$, respectively.

	Membership	Overlap	chain
		`abcdefghijk`	
a	`++-+-+-++++++`	`●      -`	1
b	`++`	`●●○`	5
c	` +-+`	`● ●○`	5
d	`   +-+`	`●  ●○`	5
e	`     +-+`	`●   ● ○`	5
f	`       +    +`	`●    ●○`	5
g	`       ++  +`	`●     ●○○ ○`	5
h	`        +-+`	`-      ●-○`	3
i	`        +++`	`●      -●○`	4
j	`          ++`	`●        ●○`	2
k	`           ++`	`●         ●`	2

	RMM	ROM
		`aBhiJ`
a	`++-+-+-++++++`	`● -`
B	`++-+-+-++  ++`	`●●○○`
h	`        +-+`	`- ●-○`
i	`        +++`	`● -●○`
J	`          +++`	`●   ●`

Algorithm 7: IdentifyLinearChains

Data: O = An adjacency list $\{a \rightarrow [b, ...]\}$ for all $a \in |elements|$ where $Overlap_{ab} > 0$
X = An adjacency list $\{a \rightarrow [b, ...]\}$ for all $a \in |elements|$ where $Overlap_{ab} = -1$
$searchOrder = order(\sum e \neq 0 \text{ for } e \in elements)$
Result: $chains$: List of length $|elements|$ of chain ID numbers

```
begin
    CO ← An empty adjacency list
    CX ← An empty adjacency list
    vertices ← |O|
    visited ← Empty boolean array of length vertices
    component ← −1 integer array of length vertices
    chainCount ← 0
    for v ∈ searchOrder do
        Visit(v)

method Visit(v):
    visited_v ← True
    for w ∈ O[v] do
        if not visited_w then
            Visit(w)
    outgroups ← unique(chain_O[v])
    outgroups.remove(−1)
    for out ∈ outgroups do
        if O[v] ⊂ CO[out] and X[v] = CX[out] then
            chain_v ← out
            CO[out].add(v)
            return
    chainCount+ = 1
    chain_v ← chainCount
    CO[chainCount] ← {O[v], v]}
    CX[chainCount] ← X[v]
```

Algorithm 8: CollapseLinearChains

Data: $Membership$ = Output from **CalculateMembershipMatrix**
$chains$ = Output from **IdentifyLinearChains**
Result: RMM : Reduced Membership Matrix of shape $|chains| \times |frags| + 4$

```
begin
    RMM ← copy(Membership)
    chainIndices ← An empty list
    chainDict ← An empty dict
    for i ∈ |vertices| do
        if chain_i ∈ keys(chainDict) then
            parent ← chainDict[chain_i]
            newInfo ← where(Membership[i, :] ≠ 0
            RMM[parent, newInfo] ← Membership[i, newInfo]
        else
            chainIndices.append(i) chainDict[chain_i] ← i
    return RMM[chainIndices, :]
```

Generate Overlap Graph

With matrices of $Membership$, $Overlap$, $Weights$ calculated for all *chains*, it is now possible to construct the Overlap Graph where $Node$ objects are assembled into $Paths$. A $Node$ object is constructed for each *chain*, and $Paths$ across multiple *chains* can also be expressed as a $Node$:

$Node$ attributes:

name	data type	description
index	*int*	Ordered ID number
length	*int*	Total exonic length (nucleotides)
IC	*int*	Information Content, $\sum info \neq 0$
maxIC	*int*	$\|frags\| + 4$
left	*int*	Lowest *index* of contained $Nodes$
right	*int*	Highest *index* of contained $Nodes$
members	*set*	$f \in frag$ where $membership_f = 1$
nonmembers	*set*	$f \in frag$ where $membership_f = -1$
LM	*int*	$min(members)$
RM	*int*	$max(members)$
strand	$\{-1, 0, 1\}$	Alignment strand
bases	*float*	Total sequenced bases contained in $Node$
cov	*float*	$bases/length$
weights	*array*	$\|sources\|$, cov contributed by each source
member_weights	*array*	$\|frags\|$, estimated cov of each $frag$
ingroup	*set*	$\{i \in \|Nodes\|$ where $Overlap_{i,Node.index} = 1\}$
outgroup	*set*	$\{i \in \|Nodes\|$ where $Overlap_{Node.index,i} = 1\}$
contains	*set*	$\{i \in \|Nodes\|$ where $Overlap_{i,Node.index} = 2\}$
contained	*set*	$\{i \in \|Nodes\|$ where $Overlap_{Node.index,i} = 2\}$
excludes	*set*	$\{i \in \|Nodes\|$ where $Overlap_{Node.index,i} = -1\}$
includes	*set*	Set of indices of $Nodes$ included in $Path$
assigned_to	*set*	Set of $Paths$ this $Node$ is part of
complete	*bool*	This $Node$ is a full-length, gapless transcript
s_tag	*bool*	This $Node$ has a Start Tag
e_tag	*bool*	This $Node$ has an End Tag
is_spliced	*bool*	This $Node$ contains at least one intron
has_gaps	*bool*	This $Node$ contains at least one internal gap

The set of $Nodes$ generated for the Overlap Graph are *elementNodes*; they map 1-to-1 to the rows of the Reduced Membership Matrix. New $Node$ objects can be constructed by combining multiple *elementNodes*, and any valid set of *elementNodes* connected by edges in the Overlap Matrix with no incompatibilities is a *Path*.

Algorithm 9: OverlapGraph Constructor

Data: M = Reduced Membership Matrix, shape $|chains| \times |frags| + 4$
O = Reduced Overlap Matrix, shape $|chains| \times |chains|$
$sourceWeights$ = Matrix recording sequenced $bases/length$, shape $|chains| \times |sources|$
$fragWeights$ = Matrix estimating coverage by $frag$ for each *chain*, shape $|chains| \times |frags|$
Result: *OverlapGraph* object: Container for the lists of *elementNodes* and *Paths*

```
begin
    OG ← An empty OverlapGraph object
    OG.elementNodes ← An empty list
    OG.Paths ← An empty list
    for i ∈ |chains| do
        OG.elementNodes.append(Node(M[i,:], O[i,], sourceWeights[i,:], fragWeights[i,:]))
        if OG.elementNodes_i.complete then
            OG.Paths.append(copy(OG.elementNodes_i))
    OG.bases ← ∑(e.bases for e ∈ OG.elementNodes)
    return OG
```

Resolve Containment

When the *OverlapGraph* object is constructed, there will usually be a number of *Nodes* that are contained by other *Nodes*. A contained *Node* is a subset of one or more containers, and assembly performance is improved the weight of contained *Nodes* is passed proportionally to its containers prior to calculating *Paths*. The Resolve Containment algorithm "bubbles up" weight from contained *Nodes* in order of decreasing information content (longest first). The total weight of the Locus is preserved, but the weights of all contained elements that cannot form a *Path* incompatible with their containers are set to zero. This allows the shorter *elementNodes* to be ignored during assembly without losing their coverage information.

Algorithm 10: ResolveContainment

```
Data: OG = Overlap Graph Object
Result: In-place update of weights in OG.elementNodes
begin
    zeros ← Set of all OG.elementNodes with cov = 0
    containedNodes ← which |e.contained| > 0 for e ∈ OG.elementNodes
    resolveOrder ← order(containedNodes, key1 = IC, key2 = |contained|)
    for i ∈ resolveOrder do
        element = OG.elementNodes_i
        if element.cov > 0 then
            containers ← element.contained \ zeros
            incompatible ← set(0 : |OG.elementNodes|) \ zeros
            for c ∈ containers do
                incompatible ← incompatible ⋂ OG.elementNodes_c.excludes
            incompatible ← incompatible \ element.excludes
            if |incompatible| = 0 then
                containerCov ← Array of OG.elementNodes_c.cov for c ∈ containers
                totalCov ← ∑ containerCov
                defaultProportions ← Array length |containers| of containerCov/totalCov
                proportions ← 0-array shape |containers| × |sources|
                for i ∈ 0 : |containers| do
                    c ← containers_i
                    weights ← OG.elementNodes_c.weights
                    proportions[i, :] ← defaultProportions × weights/ ∑ weights
                for i ∈ 0 : |sources| do
                    if ∑ proportions[:, i] = 0 then
                        proportions[:, i] ← defaultProportions
                    else
                        proportions[:, i] ← proportions[:, i]/ ∑ proportions[:, i]
                for i ∈ 0 : |containers| do
                    c ← containers_i
                    cNode ← OG.elementNodes_c
                    cNode.weights.add(element.weights × proportions[i, :])
                element.weights ← 0-array of length |sources|
                zeros.add(i)
```

Greedy Paths

The Overlap Graph can be understood to have a $source(s)$ and $sink(t)$, and regardless of alignment strand, all edges between $Nodes$ describe flow from left to right in genomic positions. The four Tag types connect to the $source(Start+, End-)$ and $sink(Start-, End+)$, so any complete $s \to t$ Path must contain a same-stranded pair of Start/End Tags. To find an optimal path through the OverlapGraph, search begins at the $Node$ with the greatest weight, and examines each edge to continue outward in a Breadth-First Search that always traverses the edge with the highest "Extension Score". This score is an estimate of the total available weight along the extending edge, counterbalanced by 3 separate penalties:

$sourceSimilarity$: [0-1] Distance between the relative source contributions of the Path and the Extension.
$variancePenalty$: [0-1] Ratio between the mean sectional coverage and max sectional coverage of the Path.
$deadEndPenalty$: [0-1] Multiplier imposed if no complete $Paths$ can extend through this edge.

When the search terminates, the resulting set of $Nodes$ is combined to yield an "Optimal Path", and is stored in a list of $Paths$. Assembly of the locus is complete when less than `--min_proportion` of the reads are unassigned or the same path is generated twice.

Algorithm 11: ExtensionScore

```
Data: Path = A Node object representing an incomplete set of elementNodes
extension = A candidate list of elementNodes to add to Path
p = minimum proportion
Result: score : Float that evaluates the extension to Path (higher is score better)
begin
    extensionWeights ← 0-array of length |frags|
    sourceProportions ← 0-array of length |sources|
    newFrags ← An empty set
    for node ∈ extension do
        newFrags.add(frags in node and not in Path)
        newProportions ← Array of length |sources|,
            { 1-array if node is unassigned
            { otherwise Path.weights/(Path.weights + ∑(p.weights for p ∈ node.assigned_to))
        available ← ∑ newProportions/|newProportions|
        if available < p then
            available ← 0
        extensionWeights.add(available ∗ node.member_weights)
        sourceProportions.add(newProportions/|extension|)
    extensionCov ← max(extensionWeights[newFrags])
    combinedWeights ← Path.member_weights + extensionWeights
    pathProportions ← Path.weights/|Path.weights|
    sourceSimilarity ← .5 ∗ (2 − ∑(abs(pathProportions − sourceProportions)))
    variancePenalty ← mean(combinedWeights)/max(combinedWeights)
    deadEndPenalty ← { 0 if s or t are unreachable from Path+extension
                     { 1 otherwise
    score ← extensionCov ∗ sourceSimilarity ∗ variancePenalty ∗ deadEndPenalty
    return score
```

Using default arguments for assembly, $deadEndPenalty$ is absolute and yields a $score$ of 0 for any $extension$ to a $Path$ that cannot be part of an $s \to t$ Path. If assembly is run with the argument `--allow_incomplete`, the penalty is instead a multiplier of .1 if s is unreachable, and a second .1 multiplier if t is unreachable. Complete $Paths$ are still heavily favored, but a $Path$ will nonetheless be produced if $s \to t$ Paths do not exist or are extremely poor. Each step of the Greedy Paths algorithm begins by generating a set of possible extensions. Each $extension$ within $extensions$ is a set of mutually compatible $elementNodes$ that can extend the given $Path$ exactly one step to the left and/or right. Each $extension$ is composed of an ingroup (a node with an edge to $Path$) and an outgroup (a node that $Path$ has an edge to), and any nodes that may be contained by the union of the ingroup, outgroup, and $Path$. The Greedy Path algorithm begins at the heaviest unassigned $elementNode$, and each extension step lengthens the $Path$ toward both the source and sink.

Algorithm 12: GenerateExtensions

Data: OG = Overlap Graph object
$Path$ = An incomplete merged set of $elementNodes$
Result: $extensions$: A list of candidate sets of $elementNodes$ to extend $Path$
begin
    $extensions \leftarrow$ An empty list of sets
    $ingroup \leftarrow Path.ingroup \bigcup Path.contained$
    $outgroup \leftarrow Path.outgroup \bigcup Path.contained$
    $freeNodes \leftarrow Path.contains \setminus Path.includes$
    if $|freeNodes| > 0$ **then**
        $Path.extend(freeNodes)$
        $ingroup \leftarrow Path.ingroup$
        $outgroup \leftarrow Path.outgroup$
    if $|ingroup \setminus Path.contained| > 0$ **then**
        if $|outgroup \setminus Path.contained| > 0$ **then**
            $pairs \leftarrow$ List of all (i, o) pairs for $i \in ingroup, o \in outgroup$ if $Overlap[i, o] > -1$
        else
            $pairs \leftarrow$ List $(i, Path.index)$ for $i \in ingroup$
    else
        $pairs \leftarrow$ List $(Path.index, o)$ for $o \in outgroup$
    for $(in, out) \in pairs$ **do**
        $e_{in} \leftarrow OG.elementNodes_{in}$
        $e_{out} \leftarrow OG.elementNodes_{out}$
        $contained \leftarrow e_{in}.outgroup \bigcup e_{out}.ingroup \bigcup e_{in}.contains \bigcup e_{out}.contains$
        `/* Filter 1: All elements already included or excluded in the extension */`
        $exclude \leftarrow e_{in}.includes|Path.includes|e_{out}.includes|e_{in}.excludes|Path.excludes|e_{out}.excludes$
        $contained \leftarrow contained \setminus exclude$
        `/* Filter 2: All elements that add information not contained in the extension */`
        $stranded \leftarrow e_{in}.strand \neq 0$ or $e_{out}.strand \neq 0$ or $Path.strand \neq 0$
        $extMembers \leftarrow e_{in}.members \bigcup Path.members \bigcup e_{out}.members$
        $extNonmembers \leftarrow e_{in}.nonmembers \bigcup Path.nonmembers \bigcup e_{out}.nonmembers$
        $exclude \leftarrow$ Set of $contained$ elements if not $contained.members \subset extMembers$ or not $contained.nonmembers \subset extNonmembers$
        $contained.add(in, out)$
        $contained \leftarrow contained \setminus exclude$
        $extensions.add(contained)$
    return $extensions$

Algorithm 13: GreedyPaths

Data: OG = Overlap Graph object
p = minimum proportion
Result: $Path$: The highest-scoring complete $s \rightarrow t$ path through the heaviest unassigned $elementNode$
begin
    $Path \leftarrow node$ with $max(node.cov$ for $node \in OG.elementNodes)$ where $|node.assignments| = 0$
    $Path.extend(Path.contains)$
    $extensions \leftarrow$**GenerateExtensions(Path)**
    while $|extensions| > 0$ **do**
        if $|extensions| = 1$ **then**
            $Path.extend(extensions_0)$
        else
            $bestExtension \leftarrow ext \in extensions$ with $max($**ExtensionScore(Path, ext)**$)$
            $Path.extend(bestExtension)$
        $extensions \leftarrow$**GenerateExtensions(Path)**
    return $Path$

Assign Weights to Paths

If an OverlapGraph has one or more *Paths*, the weights of individual *Nodes* must be assigned proportionally to the *Paths* of which they are parts. A *Node* assigned to a single *Path* assigns all its weight to that *Path*, but if multiple overlapping *Paths* exist, weight must be assigned proportionally to each. The estimated weight of each *Path* is calculated by using the previous *Path* weights as priors. *Nodes* are resolved in increasing order of number of assignments, and their weight is added proportionally to the priors of all assigned *Paths*.

Algorithm 14: AssignWeightsToPaths

Data: OG = Overlap Graph object
Result: In-place update of $weights$ in $OG.Paths$
begin
  $priors \leftarrow$ 0-array of shape $|OG.Paths| \times |sources|$
  for $i \in 0 : |OG.paths|$ **do**
    $priors[i, :] \leftarrow OG.Paths_i.weights$
    $OG.Paths_i.weights \leftarrow 0 - arrayoflength|sources|$
  $pathCovs \leftarrow \sum_{j=0}^{|sources|} priors_{ij}$
  $sampleCovs \leftarrow \sum_{i=0}^{|OG.Paths|} priors_{ij}$
  $pathProportions \leftarrow pathCovs / \sum pathCovs$
  $proportions \leftarrow$ Array shape $|OG.Paths| \times |sources|$ column-filled with $pathCovs$
  for i $where$ $sampleCovs > 0$ **do**
    $proportions[:, i] \leftarrow priors[:, i] / sampleCovs_i$
  for $i \in order(OG.assignments)$ **do**
    $element \leftarrow OG.elementNodes_i$
    if $OG.assignments_i = 1$ **then**
      $OG.Paths_{element.assigned\_to}.weight.add(element.weights)$
    else if $OG.assignments_i > 1$ **then**
      `/* Assigned paths must compete for` $element.weight$ `*/`
      $assigned \leftarrow element.assigned\_to$
      $assignedProportions \leftarrow proportions[assigned, :]$
      for $jin0 : |assigned|$ **do**
        $Path \leftarrow OG.Paths_{assigned_j}$
        $Path.weight.add(element.weight * element.length * assignedProportions[j, :])$

Path Filtering

For a signal threshold p, assembly is complete after $> (1-p)$ bases in the Overlap Graph have been assigned to *Paths*. However, due to incomplete or error-prone data, the optimal set of *Paths* may still contain a number of errors that should be removed prior to exporting the remaining *Paths* and transcript models. Five types of possible error are identified in the order listed below and are either removed or subject to more stringent filters.

Incomplete assemblies: Any *Path* that has gaps or is missing a Start or End Tag.
Fused assemblies: A *Path* that fully contains two non-overlapping *Paths*.
Truncated assemblies: A *Path* fully contained within a longer path.
Retained introns: An overlapping *Path* has a superset of introns.
Minimum isoform proportion: A *Path* is assigned $< p$ of the summed bases of overlapping *Paths*

The definition of a complete *Path* is built into the *Node* object, but the other filters need to be calculated with reference to other *Paths* in the Overlap Graph. Each filter defines a subset of *Paths* to keep, and in the *FilterPaths* method weights are reassigned after each filter because the total number of *Paths* may have changed.

Algorithm 15: FilterFusions

```
Data: OG = Overlap Graph object
Result: filteredPaths : Subset of OG.Paths that passed the filter
begin
    badPaths ← Empty boolean array length |OG.Paths|
    for i ∈ |OG.Paths| do
        p1 ← OG.Paths_i
        containedRanges ← An empty list
        for j ∈ |OG.Paths| ≠ i do
            p2 ← OG.Paths_j
            if p2.members ⊂ p1.members and p2.weight ≥ p1.weight then
                containedRanges.append((p2.LM, p2.RM))
        for c1 ∈ containedRanges do
            for c2 ∈ containedRanges do
                if c1_0 > c2_1 or c2_0 > c1_1 then
                    badPaths_i ← True
    filteredPaths ← OG.Paths[!badPaths]
    return filteredPaths
```

Algorithm 16: FilterTruncations

```
Data: OG = Overlap Graph object
Result: filteredPaths : Subset of OG.Paths that passed the filter
begin
    badPaths ← Empty boolean array length |OG.Paths|
    for i ∈ |OG.Paths| do
        containerCov ← 0
        p1 ← OG.Paths_i
        for j ∈ |OG.Paths| do
            p2 ← OG.Paths_j
            if p1 overlaps p2 and p1.members ⊂ p2.members then
                containerCov.add(p2.cov)
        if containerCov > 0 and p1.cov < p1.cov + containerCov then
            badPaths_i ← True
    filteredPaths ← OG.Paths[!badPaths]
    return filteredPaths
```

Algorithm 17: FilterRetainedIntrons

Data: OG = Overlap Graph object
$intronFilter$ = Minimum proportion of coverage to keep a retained intron
Result: $filteredPaths$: Subset of $OG.Paths$ that passed the filter
begin
    $badPaths \leftarrow$ Empty boolean array length $|OG.Paths|$
    for $i \in |OG.Paths|$ **do**
        $containerCov \leftarrow 0$
        $p1 \leftarrow OG.Paths_i$
        for $j \in |OG.Paths|$ **do**
            $p2 \leftarrow OG.Paths_j$
            if $p1.LM = p2.LM$ *and* $p1.RM = p2.RM$ **then**
                if $introns(p1) \subset introns(p2)$ **then**
                    $containerCov.add(p2.cov)$
        if $containerCov > 0$ *and* $p1.cov < intronFilter * (p1.cov + containerCov)$ **then**
            $badPaths_i \leftarrow True$
    $filteredPaths \leftarrow OG.Paths[!badPaths]$
    return $filteredPaths$

Algorithm 18: FilterMinimumProportion

Data: OG = Overlap Graph object
p = Minimum proportion
Result: $filteredPaths$: Subset of $OG.Paths$ that passed the filter
begin
    $badPaths \leftarrow$ Empty boolean array length $|OG.Paths|$
    for $i \in |OG.Paths|$ **do**
        $overlapCov \leftarrow 0$
        $p1 \leftarrow OG.Paths_i$
        for $j \in |OG.Paths|$ **do**
            $p2 \leftarrow OG.Paths_j$
            if $|p1.members \bigcap p2.members| \geq .5 * min(|p1.members|, |p2.members|)$ **then**
                $overlapCov.add(p2.cov)$
        if $overlapCov > 0$ *and* $p1.cov < p * (p1.cov + overlapCov)$ **then**
            $badPaths_i \leftarrow True$
    $filteredPaths \leftarrow OG.Paths[!badPaths]$
    return $filteredPaths$

Algorithm 19: FilterPaths

Data: OG = Overlap Graph object with a complete set of Optimal Paths
Result: In-place update of $OG.Paths$ to retain only unfiltered $Paths$
begin
    $OG.Paths \leftarrow p \in OG.Paths$ if $p.complete$
    AssignWeightsToPaths(OG)
    $OG.Paths \leftarrow$ **FilterFusions(OG)**
    AssignWeightsToPaths(OG)
    $OG.Paths \leftarrow$ **FilterTruncations(OG)**
    AssignWeightsToPaths(OG)
    $OG.Paths \leftarrow$ **FilterRetainedIntrons(OG)**
    AssignWeightsToPaths(OG)
    $OG.Paths \leftarrow$ **FilterMinimumProportion(OG)**
    AssignWeightsToPaths(OG)
