# Supplementary material for: Bookend: precise transcript reconstruction with end-guided assembly
Source: Genome Biol. 2022 Jun 29;23:143. doi: 10.1186/s13059-022-02700-3 (PMC9245221; doi:10.1186/s13059-022-02700-3)
Supplement: Supplementary file 3 — Additional file 3. Bookend user guide: Detailed guidelines and instructions for using all utilities in the Bookend software package. [file 13059_2022_2700_MOESM3_ESM.pdf]

Bookend User Guide

Michael A. Schon

v1.0.1, April 2022

1 Overview

Bookend is a transcript assembler that centralizes the importance of information about RNA 5′ and 3′ ends, in addition to splice junctions. This document describes the basic workflow and algorithms behind "end-guided assembly" used in the Bookend package.

Bookend uses general algorithms that can work with any type of RNA-seq data, but the best results will be achieved if the set of experiments contains at least one source of 5′-end sequencing and 3′-end sequencing. This can be achieved through "hybrid assembly" of standard RNA-seq, CAGE, and 3P-seq, for example, or from a long-read sequencing method such as PacBio Iso-seq. 5′-end and 3′-end information can also be extracted directly from reads of "full-length sequencing methods", such as those of the Smart-seq family optimized for single-cell sequencing. See the subcommand `bookend label` for information on extracting end-labeled reads.

If Python 3.6+ is installed in your environment, the Bookend toolkit can be installed with the command:
`pip install bookend-rna`

Bookend can also be built from source code available at
`https://github.com/Gregor-Mendel-Institute/bookend`

2 The Bookend Workflow

As a reference-guided assembler, Bookend tools are written to both pre-process and post-process RNA-seq reads aligned to a reference genome. STAR is the recommended aligner for short reads, and minimap2 for long reads. The basic workflow from raw FASTQ files to finished assembly is below. Steps followed by * are optional, depending on the RNA-seq data type and the specific use case:

```
label* -> [align] -> elr -> condense** -> assemble
*for reads with embedded end labels, e.g.  Smart-seq
**for many-sample assemblies, e.g.  single cell RNA-seq
```

These steps can be run via the main Bookend subcommands, namely `bookend label`, `bookend elr`, `bookend condense`, and `bookend assemble`. Each subcommand is described below. In addition to the main subcommands, Bookend includes a number of utilities for converting files and evaluating results. These additional subcommands include:

- `classify`
- `bedgraph`
- `fasta`
- `bed-to-elr`
- `elr-combine`
- `elr-sort`
- `elr-subset`
- `elr-to-bed`
- `gtf-to-bed`
- `gtf-ends`
- `sam-to-sj`
- `sj-merge`
- `sj-to-bed`

3 Bookend subcommands

3.1 Label: Trim and label End Tags from FASTQ files.

usage: `bookend label [options] FASTQ1 [FASTQ2]`

Positional arguments:		
FASTQ	(required)	Input FASTQ file(s). 1 for single-end, 2 for paired-end.
Other arguments:	Default	Description
-S|--start [str]	AAGCAGTGGTATCAACGCAGAGTACATGGG	Start Tag primer sequence ($5'$ end, e.g. Smart-seq TSO)
-E|--end [str]	ACGCAGAGTACTTTTTTTTTTTTTTTTTTTT+	End Tag primer sequence ($3'$ end, e.g. oligo-dT)
--umi {S,E}	(None)	The Start or End tag contains a UMI to record (string of N's)
--strand {forward, reverse, unstranded}	unstranded	Strandedness of equencing method
--out1 [file]	label.1.fq	Filepath to write Mate 1 output (paired-end)
--out2 [file]	label.2.fq	Filepath to write Mate 2 output (paired-end)
--single_out [file]	label.single.fq	Filepath for single-end output
--min_start [int]	7	Minimum allowed match to Start Tag
--min_end [int]	9	Minimum allowed match to End Tag
--mismatch_rate [0-1]	0.06	Permit up to this percent of mismatches in the tag match
--minlen [int]	18	Discard trimmed reads shorter than this
--minqual [float]	16	Discard any reads with a mean phred score lower than this
--qualmask [float]	25	Set low-quality basecalls to N for more lenient matching
--discard_untrimmed	False	Do not keep reads without a trimmed tag
--pseudomates	False	Pipe single reads to --out1 with an artificial mate pair in --out2 (overrides --single_out)

From one single-end FASTQ file or two paired-end FASTQ files, `bookend label` scans each read for a matching "Start Tag" and/or "End Tag", then outputs between one and three trimmed FASTQ files with the type and length of end labels appended to the read name as a "_TAG=" suffix. All labeled reads are reoriented so that Mate 1 is sense to the original RNA strand. The Start Tag sequence can be provided with the `-S` argument, and should be the sense-stranded sequence of the oligo in cDNA upstream of $5'$ ends. For Smart-seq methods this is the Template Switching Oligo (TSO). The End Tag is the sequence of the $3'$ oligo used during reverse transcription, usually an oligo-d(T) primer. To trim tags of uncertain length, a "+" can be added after the last nucleotide, and this will be treated as an oligomer of indefinite length. For example, passing the argument `-E TTTTTTTTTT+` will cause `bookend label` to trim and label all sequences of 10 or more T's/A's. For reads that contain a Unique Molecular Identifier (UMI) within a Start Tag or End Tag, the `--umi` argument specifies where to look for the UMI sequence, which will be appended as an additional "_UMI=" suffix. For oligo-d(T) End Tags, it is good to provide at least part of the oligo sequence upstream of the T oligomer; this helps minimize the number of templated oligo-T/oligo-A sites that are falsely labeled as End Tags. If your files do not contain Illumina-encoded Phred quality scores, set `--minqual -1 --qualmask -1`.

3.2 ELR: Convert aligned BAM to End-Labeled Read files.

usage: `bookend elr [options] INPUT`

Positional arguments:		
INPUT	(required)	Input BAM/SAM file; must be name-sorted
**Other arguments:**	**Default**	**Description**
-o|--output [file]	[INPUT].elr	Path to write output ELR file
--source [str]	(None)	Name of the sample
--genome [file]	(None)	Path to reference genome FASTA (required for artifact masking)
--start_seq [str]	ACATGGG	Sequence to match for 5′ artifact masking
--end_seq [str]	RRRRRRRRRRRRRRR RRRRRRRRRRRRRRR	Sequence to match for 3′ artifact masking
--mismatch_rate [0-1]	0.2	Max mismatch rate for artifact masking
--stranded	False	Expect read strand to match RNA strand
-s	False	All reads are 5′-end reads
-c	False	All reads are capped 5′-end reads
-e	False	All reads are 3′-end reads
--no_ends	False	Ignore all 5′ and 3′ end labels
--bed	False	Output a 15-column BED file (BED12+3)
--secondary	False	Keep secondary alignments
--header [file]	(None)	Write ELR header to separate file
--record_artifacts	False	Add alternate >/] tags to artifact-masked S/E reads
--split	False	Write multimappers to separate files
--remove_noncanonical	False	Require canonical splice junctions (GT/AG)
--sj_shift [int]	2	"Repair" noncanonical splice junctions by shifting up to this distance
--minlen_strict	18	Keep reads this short if unique perfect matches with no softclipping
--minlen_loose	25	Keep reads this short if they pass other alignment parameters
--error_rate [0-1]	0.1	Discard exons with higher indels/mismatches per base

This is the main utility for generating End Labeled Read (ELR) files from aligned, name-sorted BAM/SAM files. This step can also filter out common sources of erroneous end labels, which is strongly recommended for accurate assemblies. Specifically, oligo-d(T) primers can bind to genome-templated A-rich sequences within the RNA molecule in addition to non-genome-templated poly(A) tails. Depending on the experiment and organism, this "oligo-dT mispriming" can be extremely common, resulting in many false positive End Tags. Various 5′-end sequencing techniques also suffer from known artifacts. Libraries that make use of Template Switching Oligos (TSO), including the Smart-seq and IsoSeq methods and some 5′-end sequencing protocols, are susceptible to template switching artifacts at regions that share sequence similarity to the TSO's 3′ end. If a reference genome is provided to `bookend elr`, then it can perform artifact masking for both 5′ and 3′ End Tags.

Artifact masking depends on the sequences provided by `--start_seq` and `--end_seq`. These two sequences should be the expected untemplated sequence on the sense cDNA strand immediately upstream and downstream of the RNA sequence, respectively. Default parameters are optimized based on Smart-seq2 libraries in mouse and Arabidopsis. These include a very permissive mismatch rate (20%), and an oligo-dT mispriming site of up to 30 purines (IUPAC code R). If a long artifact masking sequence is provided, matching is performed up to the trimmed tag length present in the read's "_TAG=" suffix.

When provided a reference genome, `bookend label` also performs Cap Inference on Start Tags. In 5′-end labeled sequencing libraries, the presence of a non-genome-templated 5′-terminal G between the trimmed oligo and the aligned sequence is evidence that this read possessed a 7_mG cap structure. These reads can be extremely valuable for distinguising genuine transcription start sites from the products of RNA degradation that occur both in vivo and in vitro.

3.2.1 The End Labeled Read file format

End Labeled Read (ELR) files are defined in two parts: a header that builds an index of reference chromosomes (#C) and read sources (#S), and a 7-column body with the following contents:

1. Chromosome index [int]
2. Alignment start position [int]
3. Alignment length (including gaps) [int]
4. Strand {+, -, .}
5. ELCIGAR string describing alignment labels and gaps [str]
6. Source of read [int]
7. Weight of reads matching this description (start—cov—end) [float]—[float]—[float]

ELCIGAR strings describe the position and labels of all aligned segments of a read, patterned off of the BAM/SAM format CIGAR strings but with additional End Label information. They are strings of Character/Number pairs with one trailing character ($[CN]_x C$), where C is a label and N is a numeric distance on the genome. Each label annotates the end of the associated span as one of the following:

S	Start Tag (RNA 5′ end)
C	Cap Tag (5′ end with untemplated G)
E	End Tag (RNA 3′ end)
D	Splice junction donor
A	Splice junction acceptor
.	Unspecified gap/end

For example, the ELCIGAR for a 50bp paired-end read of a 185nt cDNA fragment with no introns or end labels would be ".50.85.50.". A full-length transcript with the ELCIGAR "$S256D800A128D800A512E$" has 3 exons of 256, 128, and 512nt, respectively, and 2 introns that are both 800nt.

Short indels and mismatches are not recorded in ELCIGAR strings, but the number of alignment errors within each exonic region (between adjacent SD, CD, AD, and AE pairs) is tallied. If this tally exceeds `--error_rate` as a proportion of the number of aligned bases in that exon, then it is removed from the ELCIGAR and the surrounding exons are bridged with an unspecified gap (.. pair). This setting prevents the use of splice junctions from especially error-prone alignments, which can be common in some long-read sequencing protocols.

During assembly, the "weight" column will be used to determine read coverage depth for exonic frags and for end positions. It can either be a single float value (coverage), or a set of 3 floats separated by pipe symbols, indicating start tags—coverage—end tags. These values will only differ if the ELR feature is the product of assembly. If a Start Tag, Cap Tag, or End Tag is lowercase (s, c, e), `bookend assemble` will treat these ends as having a weight of 1 instead of the read weight. This can be beneficial for partial assembly (discussed in the next section) of sparsely-labeled samples.

3.3 Condense: Partial assembly

`usage: bookend condense [options] INPUT`

Positional arguments:		
INPUT	(required)	Input single sorted ELR file
Other arguments:	Default	Description
--starts	False	Sample is a Start Tag (5′-capture) file, e.g. CAGE
--ends	False	Sample is an End Tag (5′-capture) file, e.g. 3P-Seq
--sparse	False	Sample is sparsely end-labeled, e.g. Smart-seq
-o|--output [file]	[INPUT].cond.elr	Path to write output ELR file
--max_gap [int]	50	Largest tolerable gap size between reads
--end_cluster [int]	50	Merge End Tags within this distance into one cluster
--min_overhang [int]	3	Smallest exonic overhang to keep
--min_cov [float]	1	Discard partial transcripts below this coverage depth
--min_len [int]	20	Discard partial transcripts shorter than this
--min_intron_len [int]	50	Discard introns below this size
--min_proportion [0-1]	0.01	Signal threshold: discard ends, junctions, frags, or isoforms below this percentage
--intron_filter [0-1]	0.15	Coverage filter used for retained introns
--cap_bonus [float]	5	Multiply the weight of Cap Tags by this value
--cap_filter [0-1]	0.1	Require downstream start sites to contain at least this proportion of Cap Tags

This function is a special case of end-guided assembly, in which assemblies are not penalized or filtered out if they are not end-to-end complete. This ”partial assembly” is specifically designed for single-cell RNA-seq datasets, so that the reads from each cell can be condensed for efficient meta-assembly.

Partial assembly takes a single ELR file input and writes a single ELR file. By default all reads are reported with minimal filtering. All loci with multiple overlapping reads are assembled using the standard end-guided assembly algorithms, but no penalty is applied for incomplete starts/ends. These output files can then be used as input to `bookend assemble`. Assembling first on the single-cell level guarantees that exon chains from each cell remain coherent during the full-scale assembly. End-capture libraries can be condensed in a way that exclusively retains end clusters and their abundance using the argument `--start` or `--end`. For sparsely labeled samples, like short reads from full-length sequencing protocols, coverage depth of the condensed read will not be representative of of the Start Tag and End Tag abundance, so the `--sparse` argument writes end labels as lowercase to drop their priority during assembly.

3.4 Assemble: End-guided assembly/meta-assembly

usage: bookend assemble [options] INPUT

Positional arguments:		
INPUT[...]	(required)	Input one or more sorted ELR files
Other arguments:	Default	Description
-o|--output [file]	bookend_assembly.gtf	Path to write assembled output
--max_gap [int]	50	Largest tolerable gap size between reads
--cov_out [file]	None	Destination for a TSV of estimated coverage by source
--end_cluster [int]	50	Merge End Tags within this distance into one cluster
--max_gap [int]	50	Largest gap to tolerate with no coverage
--end_cluster	200	Largest distance between end-labeled reads to count in the same cluster
--min_overhang [int]	3	Smallest exonic overhang to keep
--min_cov [float]	2	Discard partial transcripts below this coverage depth
--allow_incomplete	False	Keep assemblies that are gapped or missing ends
--min_unstranded_cov [float]	20	If --allow_incomplete, coverage required for assemblies with no strand
--min_start [float]	1	Minimum read number in a $5'$ cluster
--min_end [float]	1	Minimum read number in a $3'$ cluster
--min_len [int]	50	Discard partial transcripts shorter than this
--min_intron_len [int]	50	Discard introns below this size
--min_proportion [0-1]	0.01	Signal threshold: discard ends, junctions, frags, or isoforms below this percentage
--intron_filter [0-1]	0.15	Coverage filter used for retained introns
--cap_bonus [float]	5	Multiply the weight of Cap Tags by this value
--cap_filter [0-1]	0.02	Require downstream start sites to contain at least this proportion of Cap Tags
--require_cap	False	Require all start sites to pass --cap_filter
--ignore_labels	False	Assemble without using any end label information
--use_sources	False	Maximize read source coherence during assembly

This is the main Bookend subcommand. From one or more sorted ELR files, `bookend assemble` packages reads into loci, then resolves each locus via end-guided assembly. The algorithms are more fully described as pseudocode in the manuscript appendix "Assembly Algorithms".

Assembly begins by reading through the input file(s) and packaging reads into discrete chunks. If a gap between adjacent aligned reads larger than `--max_gap` exists, the current chunk of reads is processed and a new one is started. The argument `--min_proportion` is used as a global "noise filter" and sets the threshold below which rare features should be filtered out. After filtering low-abundance splice junctions within the chunk, it can be further split into subchunks if gaps appear that were previously passed over by a filtered splice junction.

In addition to the global noise filter, the user has control over a number of other filters that can be modified to either retain or remove lower-confidence assemblies. `--min_cov`, `--min_start`, and `--min_end` set the threshold of evidence required for a transcript's estimated abundance, Start Tags, and End Tags, respectively. Limits can also be imposed on the structure of the transcript with `--min_len` to exclude very short transcripts and

--min_intron_len to ignore very short introns.

The user can also determine the characteristics of Start Tag and End Tag clustering. Both Transcription Start Sites (TSS) and Polyadenylation Sites (PAS) can occur in broad clusters, sometimes spanning hundreds of bases on the genome. The End Clustering algorithm treats Start Tags on the same strand within a distance --end_cluster to belong to the same Start Cluster, and the same is done for End Tags. Additionally, the weight of Cap Tags (Start Tags with an upstream untemplated G) is multiplied by --cap_bonus. To further minimize the chance of false positive TSS from RNA cleavage or degradation, a --cap_filter is enforced for Start Clusters not at the extreme ends of a locus. Finally, passing the argument --require_cap excludes even terminal Start Clusters if they do not pass the --cap_filter.

Finally, Bookend can source information in the edge weights of its Overlap Graph, which reduces the chances of producing chimeric transcripts with making an assembly from multiple samples. The estimate coverage of each source in each sample can be written to a TSV file if the filepath is provided to --cov_out. In some cases it may be beneficial to ignore source, like in a hybrid assembly that uses reads from multiple library types (in the manuscript this is demonstrated with short-read + long-read + $5'$-end libraries). By default assembly is "source-naive"; --use_source may improve assembly, but it can be very resource intensive if there are many input samples. If more than 50 samples are being combined, use bookend condense on each sample beforehand, then assemble the set of condensed assemblies.

3.5 Classify: Comparison of two annotation files

`usage: bookend classify [options] -r REFERENCE -i INPUT`

Required arguments:		
-r [file]	(required)	GTF/GFF3/BED12 file of reference annotations
-i [file]	(required)	Input GTF/GFF3/BED12 file of query annotations
**Other arguments:**	**Default**	**Description**
-o|--output [file]	classify.tsv	Path to write output TSV file
--end_buffer [int]	100	Distance between ends to consider a match
--ref_parent [list]	mRNA transcript	Line type(s) signifying a parent object
--ref_child [list]	exon	Line type(s) signifying a child object
--parent_attr_gene [str]	gene_id	(GTF/GFF) Attribute that stores gene_id in parent objects
--child_attr_gene [str]	gene_id	(GTF/GFF) Attribute that stores gene_id in child objects (exons)
--parent_attr_transcript [list]	transcript_id	(GTF/GFF) Attribute(s) storing transcript_id in parent objects
--child_attr_transcript [list]	transcript_id	(GTF/GFF) Attribute(s) storing transcript_id in child objects (exons)
--bed_gene_delim [str]	.	String that splits gene name from isoform number

This utility compares two annotation files; one acts as a ”reference”, and the other acts as ”input”. For each transcript model in the input annotation, the reference annotation is queried for the closest structural match. A hierarchy of classifications is used to define each input transcript:

full_match	Exact exon chain, starts and ends closer than `--end_buffer`
exon_match	Exact exon chain, but ends do not match
fusion	Shares exons with 2 or more reference genes
fragment	Contained in a reference transcript, missing exon(s)
isoform	Closest match has incompatible exon chain
intronic	Fully contained in a reference intron (sense)
antisense	Only overlaps a reference transcript on antisense strand
ambiguous	Nonstranded transcript overlaps a reference transcript
intergenic	No reference overlap

The output file is a 13-column TSV file with the following information:

assembly_id	Input transcript name
classification	Class of the reference match
ref_match	Transcript ID of the reference match
ref_gene	Gene ID of the reference match
assembly_len	Total length (nucleotides) of input transcript's exons
ref_len	Exonic length (nucleotides) of reference match
overlap_len	Exonic length of overlap between input and reference match
diff5p	Distance (nucleotides) between input Start and reference Start
diff3p	Distance (nucleotides) between input End and reference End
cov	Weight per base of input transcript
S.reads	Total weight of Start Tags in the input's Start Cluster
S.capped	Total weight of Cap Tags in the input's Start Cluster
E.reads	Total weight of End Tags in the input's End Cluster

3.6 Bedgraph: Generate coverage tracks of ELR files

usage: `bookend bedgraph [options] INPUT`

Positional arguments:		
INPUT[...]	(required)	Input one or more ELR files
Other arguments:	Default	Description
-o|--output [file]	bookend.bedgraph	Path to write output BEDGRAPH file
-t|--type {COV,5P,3P,S,E,C}	COV	Type of feature to sum in bedgraph
-s|--strand {.,+,-}	.	Output strand; default . (both strands)

This utility compares takes one or more ELR files as input and generates a bedgraph file that summarizes read abundance by genomic position. By default, the entire aligned length of all reads will be "piled up". It can be more informative to examine only the 5′ end of start-labeled reads or the 3′ end of end-labeled reads. with the `--type` argument, you can choose to quantify only the 5′- or 3′-terminal positions of starts and ends, respectively. `-t E` and `-t 3P` are identical; however, `-t 5P` counts all start-labeled reads regardless of the presence/absence of a cap, whereas `-t C` only counts caps and `-t S` only counts start tags without caps.

When counting ends, the behavior of `--strand .` changes. If no strand is specified for `-t COV`, it is assumed that read coverage is not strand-specific, and all reads will be piled up as positive values. However, if counting start tags or end tags, `--strand .` will add forward-stranded tags as positive values and reverse-stranded tags as negative values. This is a more compact representation than a separate bedgraph file for each strand, but caution! In the even of tags occupying the same genomic position on opposite strands, they will cancel each other out.

3.7 Fasta: Generate transcript FASTA files for an annotation

usage: `bookend fasta [options] --genome [file] INPUT`

Positional arguments:		
INPUT	(required)	One feature file [GTF/GFF3/BED/ELR]
Required arguments:		
--genome [file]	(required)	FASTA file of reference genome
Other arguments:	Default	Description
-o|--output [file]	bookend.fasta	Path to write output FASTA file
--allow_unstranded	False	Write unstranded transcripts to FASTA file (forward strand)

`bookend fasta` converts an annotation from a set of genomic coordinates (stored as a GTF, GFF3, BED12, or ELR file) to a set of transcript sequences in FASTA format. Introns are removed and the reverse complement is written for reverse-stranded features.

3.8 Other Utilities

3.8.1 bed-to-elr

usage: `bookend bed-to-elr [options] INPUT -o OUTPUT`

Positional arguments:		
INPUT	(required)	Input BED file
-o [file]	(required)	Output ELR file
**Other arguments:**	**Default**	**Description**
--header [file]	None	Optional separate filepath to write the ELR header
--source [str]	None	Source name of the BED file
-j	False	BED entries are splice junctions
-s	False	All BED entry $5'$ ends have Start Tags
-c	False	All BED entry $5'$ ends have Cap Tags
-e	False	All BED entry $3'$ ends have End Tags

`bookend bed-to-elr` can be used to convert any BED (5-column or 12-column) to an ELR file. The arguments `-s, -c, -e` can specify that the BED file describes $5'$ and/or $3'$ features. This allows `bookend assemble` to utilize existing TSS/PAS resources for assembly, like the FANTOM CAGE datasets.

3.8.2 elr-combine

usage: `bookend elr-combine [options] INPUTS -o OUTPUT`

Positional arguments:		
INPUT [...]	(required)	A list of input sorted ELR files
-o [file]	(required)	Output combined ELR file
**Other arguments:**	**Default**	**Description**
--temp [str]	_combinetmp	Prefix for temporary files

Combine more than one ELR file into a single position-sorted file with a uniform header. If the number of input files exceeds your open file resource limits, the files will be sent to a set of temp files with the `--temp` prefix, then these temp files will be combined.

3.8.3 elr-sort

usage: `bookend elr-sort [options] INPUT`

Positional arguments:		
INPUT	(required)	Input ELR file
**Other arguments:**	**Default**	**Description**
-o|--output [file]	stdout	Path to write sorted ELR file
-f|--force	False	Force overwrite of output file if it exists

Sorts an ELR file to guarantee that it is properly formatted for assembly.

3.8.4 elr-subset

usage: `bookend elr-subset [options] INPUT`

Positional arguments:		
INPUT	(required)	Input BAM/SAM file; must be name-sorted
-r|--region [str]	(required)	[chrom:start-end] Region to write to output
**Other arguments:**	**Default**	**Description**
-o|--output [file]	stdout	Path to write output ELR file
-f|--force	False	Force overwrite of output file if it exists

Write a smaller ELR that only contains reads overlapping the specified genomic range.

3.8.5 elr-to-bed

usage: `bookend elr-to-bed [options] INPUT -o OUTPUT`

Positional arguments:		
INPUT	(required)	Input ELR file
-o [file]	(required)	Output BED file
Other arguments:	Default	Description
--header [file]	None	Optional separate filepath to write the ELR header

Converts an ELR file to a BED12 file. 3 extra columns are appended to the end of the file (BED12+3) to record the read weight, source, and ELCIGAR, in that order.

3.8.6 gtf-to-bed

usage: `bookend gtf-to-bed [options] INPUT`

Positional arguments:		
INPUT	(required)	Input BAM/SAM file; must be name-sorted
Other arguments:	Default	Description
-o|--output [file]	stdout	Path to write output BED12 file
-f|--force	False	Force overwrite of output file if it exists
--name [str]	transcript_id	GTF attribute to pass to the BED name column
--score [str]	None	GTF attribute to pass to the BED score column
--gtf_parent [list]	transcript	Line type(s) in GTF file that define a Parent object
--gtf_child [list]	exon	Line type(s) in GTF file that define a Child object
--gff_parent [list]	mRNA transcript	Line type(s) in GFF3 file that define a Parent object
--gff_child [list]	exon	Line type(s) in GFF3 file that define a Child object
--child_attr_gene [str]	gene_id	(GTF/GFF) Attribute that stores gene_id in child objects (exons)
--parent_attr_transcript [list]	transcript_id	(GTF/GFF) Attribute(s) storing transcript_id in parent objects
--child_attr_transcript [list]	transcript_id	(GTF/GFF) Attribute(s) storing transcript_id in child objects (exons)
--color_code [file]	None	Tab-separated file of transcript types -> R,G,B colors
--color_key [str]	None	GTF/GFF3 attribute name to lookup transcript type

Converts a GTF/GFF3 file to BED12. This can be used, for example, to convert an annotation file into a format that can be used in assembly. It is also recommended to convert GTF or GFF3 annotations for `bookend classify`, since these file formats can have inconsistent formatting that results in improper parsing of the transcript models.

3.8.7 gtf-ends

`usage: bookend gtf-ends [options] INPUT`

Positional arguments:		
INPUT	(required)	Input position-sorted GTF/GFF3/BED file
**Other arguments:**	**Default**	**Description**
--extend [int]	0	Extend each feature a fixed distance up and down from the peak position
-t|--type {all,S,E}	all	Write end type: Starts (S), Ends (E), or both.
-o|--output [file]	stdout	Path to write output BED file
-f|--force	False	Force overwrite of output file if it exists
--name [str]	transcript_id	GTF attribute to pass to the BED name column
--score [str]	None	GTF attribute to pass to the BED score column
--gtf_parent [list]	transcript	Line type(s) in GTF file that define a Parent object
--gtf_child [list]	exon	Line type(s) in GTF file that define a Child object
--gff_parent [list]	mRNA transcript	Line type(s) in GFF3 file that define a Parent object
--gff_child [list]	exon	Line type(s) in GFF3 file that define a Child object
--child_attr_gene [str]	gene_id	(GTF/GFF) Attribute that stores gene_id in child objects (exons)
--parent_attr_transcript [list]	transcript_id	(GTF/GFF) Attribute(s) storing transcript_id in parent objects
--child_attr_transcript [list]	transcript_id	(GTF/GFF) Attribute(s) storing transcript_id in child objects (exons)

Iterates over every locus with one or more features, and reports the unique set of 5′ and/or 3′ ends in a BED file. Features that share the same end (or up to `--extend` distance away) are collapsed into a single end cluster, and the name of each feature belonging to an end cluster is saved as a comma-separated list in the end cluster's name column. The name is additionally give a 2-letter prefix: S/E for start or end, and P/M for alignment to the plus or minus strand. For example, if a Bookend assembly is sorted into a reference annotation, an end cluster name might look like:

"SP:Reference.01110.1,bookend.46.1,bookend.46.2"

This end cluster was a plus-stranded Start site that belonged to one reference transcript (named Reference.0110.1) and two bookend assemblies (bookend.46.1 and bookend.46.2).

3.8.8 sam-to-sj

usage: bookend elr [options] INPUT

Positional arguments:		
INPUT	(required)	Input BAM/SAM file; must be name-sorted
-F|--fasta	(required)	Genome FASTA file
Other arguments:	Default	Description
--format star,bed	star	Output file format
--filter	False	Remove noncanonical splice junctions from the output

From an aligned SAM/BAM file, writes an SJ.out.tab file containing all splice junctions. Output can be either in the STAR format or standard BED format. The provided genome FASTA file is used to identify and filter out noncanonical splice junctions.

3.8.9 sj-merge

usage: bookend elr [options] INPUT

Positional arguments:		
INPUT [...]	(required)	Multiple input SJ.out.tab files
Other arguments:	Default	Description
-o|--output [file]	sj_merge.out.tab	Filepath to write merged file
--format star, bed	star	Output file format
--min_unique [int]	0	Filter SJs with fewer unique reads
--min_reps [int]	1	Filter SJs detected in fewer than this many files
--new	False	Keep only SJs not present in the reference SJDB

For STAR SJ.out.tab files. Combine multiple SJ files into one, and apply optional filters. This utility can be used to create a high-confidence SJDB file from first-pass-aligned reads to improve sensitivity of novel splice junctions during second-pass alignment.

3.8.10 sj-to-bed

usage: bookend elr [options] INPUT

Positional arguments:		
INPUT	(required)	Input SJ.out.tab file
Other arguments:	Default	Description
-o|--output [file]	SJ.bed	Path to write output BED file

Some aligners may ask for an SJDB file in BED format, like minimap2. This utility converts the STAR SJ.out.tab format to a standard 5-column BED file.
