# Supplementary material for: Bookend: precise transcript reconstruction with end-guided assembly
Source: Genome Biol. 2022 Jun 29;23:143. doi: 10.1186/s13059-022-02700-3 (PMC9245221; doi:10.1186/s13059-022-02700-3)
Supplement: Supplementary file 4 — Additional file 4. Review history. [file 13059_2022_2700_MOESM4_ESM.docx]

**1^st^ round**

**Reviewer 1**

Authors presented an end-guided transcript assembly software package Bookend by integrating RNA 5′ and 3′ ends into the procedure, that is able to incorporate data from different RNA-seq techniques. They demonstrated with Bookend that correct modeling of transcript start and end sites is essential for precise transcript assembly, and that utilization of end-labeled reads can improve the precision of transcript assembly. To the best of my knowledge, this is the first transcript assembly algorithm that integrates RNA 5′ and 3′ ends into its procedure. I agree with that the improvement of precision is mainly caused by that integration. In my opinion, apart from the integration of the RNA 5′ and 3′ ends, the Bookend could be similar to TranComb [1] and TransLiG [2] in their precision. Methodologically, it is interesting to construct the overlap graph by introducing the membership matrix. The method design is sound to the aims of the study, and well described. The conclusions are adequately supported by the data shown. But then I have concerns below:

1. In Line 595, According to the definition of weight on nodes, "maximal similarity of weight distribution across samples between element and extension" should be changed to "maximal similarity of coverage distribution across samples between element and extension" .

2. Using example to explain why clusters are not allowed to span same-stranded spice junctions. For same-stranded Start Clusters and End Clusters that overlap, please authors explain how you further subdivided to prevent malformed transcripts.

3. In calculate Membership Matrix section in supplementary materials, please authors explain what they mean by readBases and memberBases in ΣreadBasesΣmemberBases, and over what Σ takes.

4. In Greedy Path section, in the statement "Assembly of ...... or the same path is generated twice." Please authors explain why the same path can be generated twice.

5. Although the method seams to be interesting, the computational complexity is higher. The authors should compare Bookend's running time to the previous of same kind such as [1] and [2] mentioned above.

6. In main text, are there typos in the expressions µg, ng and ug?

[1] TransComb: genome-guided transcriptome assembly via combing junctions in splicing graphs, Genome Biology (2016)

[2] TransLiG: a de novo transcriptome assembler that uses line graph iteration, Genome Biology (2019)

**Reviewer 2**

The manuscript entitled "Precise Transcript Reconstruction with End-Guided Assembly" describes a new genome-guided transcript assembler called Bookend. Although there are already a number of RNA-Seq assembly methods, Bookend's value is in using transcript start and end information which is embedded in the read data for more precise transcript assembly. Its other novelty is the ability to work with RNA sequencing data from many different platforms: short, long and end capture.

I found the manuscript very well prepared; it was written well with appropriate validation and figures. The first author has done an outstanding job in describing the software in supplementary material and the online user guide. I found the software to be very user friendly. This is often overlooked and I was pleased to see the effort that was put into this aspect of the work. Using the transcript end information is a nice idea and it appears to provide more refined annotation, which is valuable. However transcript end information is only available from particular sequencing protocols such as Smart-seq and PacBio, which is a limitation. My major comments center on clarifying this limitation in more detail in the text of the manuscript.

Major comments:

1) Although the dependence on end information is discussed throughout the manuscript, I felt that this needed to be stated upfront and more clearly. Specifically: i) In the introduction and abstract: "with a focus on identifying and utilizing RNA 5' and 3' ends." and "utilizing RNA 5' and 3' end information contained in RNA-seq dataset", it should be clarified that end information refers to the oligo sequences in the read data, or this could be misunderstood (e.g. to mean sets of heuristics based on things like coverage drops) ii) the introduction could be improved by describing which sequencing technologies do and don't provide end information. iii) In the results: stating explicitly that Bookend labels the reads by looking for particular oligo sequences which are platform dependent. Also saying that bookend can be run without end labels if desired. In this case how does the algorithm work without the S and E nodes in Fig. 1?

2) The limitations of the methods should be described. For example, without end labeled data (which is the majority of RNA sequencing data generated), it only performs with similar accuracy to existing methods like Stringtie (Fig. 2D-F). Is there a way this could be overcome. For example, is it worth a users seeking public Smart-seq or PacBio data to compliment their other datasets for use with Bookend? Even if it's from a different tissue?

Even with data that can be end-labeled, genes with low expression are unlikely to be assembled due to a lack of end reads (Fig. 2A/2C). This may explain why other assemblers produce more novel transcripts. Although the authors compare to reference annotations, these are known to be incomplete and in particular RefSeq is known to miss many transcripts reported by other annotations. Similarly, PacBio data is generally low coverage, so rare transcripts will be missed. Often the aim of transcript assembly is to improve differential expression and it is not clear how Bookends performs in this regard. Can the authors comment on this? They could also state the fraction of reads mapping to transcripts for each of the different assemblers, which is an important alternative metric to consider.

3) The computational resources required to run Bookend should be compared against alternative assemblies. Bookend does not need to be the fastest, but it should be shown that this is not a limitation to the software's use in practice.

Minor comments/questions:

1) Figure 1C - Why don't the nodes corresponding to single frag elements (e.g. 4) have incoming edges in the directed graph (e.g. 3->4 rather than 4->3)?

2) Figure 3E - If Bookend is already established as the best assembler in 3D, why not show the difference of the Bookend hybrid compared to Bookend non-hybrid? Also, is the Stringtie assembly hybrid here?

3) The explanation for read-through transcripts being removed by Bookends is the addition of end information in the assembly, but read-through transcripts should similarly have starts and ends as they are genuine transcripts. However read-through events are rare, so perhaps these are filtered out because the coverage of end-labeled reads is low?

4) The final line in "Discussion", "Bookend enables the wholesale reannotation of genomes at single-cell resolution…". I think this should be reworded. Annotations are usually considered universal (ie. non-tissue or cell specific). I think the authors are referring here to annotating the expressed transcripts at single-cell level resolution.

**Authors’ response**

Reviewer #1: Authors presented an end-guided transcript assembly software package Bookend
by integrating RNA 5′ and 3′ ends into the procedure, that is able to incorporate data from
different RNA-seq techniques. They demonstrated with Bookend that correct modeling of
transcript start and end sites is essential for precise transcript assembly, and that utilization of
end-labeled reads can improve the precision of transcript assembly. To the best of my
knowledge, this is the first transcript assembly algorithm that integrates RNA 5′ and 3′ ends into
its procedure. I agree with that the improvement of precision is mainly caused by that
integration. In my opinion, apart from the integration of the RNA 5′ and 3′ ends, the Bookend
could be similar to TranComb [1] and TransLiG [2] in their precision. Methodologically, it is
interesting to construct the overlap graph by introducing the membership matrix. The method
design is sound to the aims of the study, and well described. The conclusions are adequately
supported by the data shown. But then I have concerns below:
1. In Line 595, According to the definition of weight on nodes, "maximal similarity of weight
distribution across samples between element and extension" should be changed to "maximal
similarity of coverage distribution across samples between element and extension".
We thank the Reviewer for offering more precise wording for line 595. We have updated our
description of the Extension Score here as well as in the Greedy Paths subsection of Additional
file 1: Supporting notes.

2. Using example to explain why clusters are not allowed to span same-stranded spice
junctions. For same-stranded Start Clusters and End Clusters that overlap, please authors
explain how you further subdivided to prevent malformed transcripts.
There are multiple potential problems that arise when attempting to summarize a cluster of RNA
ends using a single consensus position. For example, transcription start sites (TSSs) may be
dispersed over hundreds of nucleotides with no single dominant peak (see Carninci et al. 2006
Nature Genetics; Zhao et al. 2011 PLoS One). Splicing can occur inside a dispersed TSS
cluster, but if the consensus position of this TSS cluster is downstream of a splice donor site,
then no valid transcript can be described which contains that splice junction. To illustrate this

point, we have attached an explanatory graph derived from Figure 1A and shown in Additional
file 1: Supporting notes, as well as an example at the 5' end of the mouse gene Actr3 from the
mESC datasets used in this manuscript.
Overlapping same-stranded clusters are a rare edge case that arise from either sequencing
artifacts or certain classes of noncoding RNA (e.g. small nucleolar RNAs). A resolution is only
necessary if the overlap contains "out-of-order peaks"; that is, the consensus Start position is
downstream of the consensus End position. If out-of-order peaks are found, the S peak position
is used to split the E cluster into two and vice versa. The method for finding and splitting these
instances, as well as the example illustrated below, has now been described as the new
Algorithm 3: ResolveOverlap in the subsection “Tag Clustering” of Additional file 1: Supporting
Notes of the revised manuscript.

3. In calculate Membership Matrix section in supplementary materials, please authors explain
what they mean by readBases and memberBases in ΣreadBasesΣmemberBases, and over
what Σ takes.
When representing an RNA-seq read in the Membership Matrix, information about precisely
where the read begins and ends is lost. For example, in Figure 1A there are 21 reads fully
contained in frag i. However, because frag i is longer than each read it contains, it would be
inaccurate to record a weight of 21 for element 11 in the Membership Matrix in Figure 1B.

Instead, the weight of element 11 is calculated as the total number of nucleotides covered by the
element (ΣreadBases) divided by the total number of nucleotides covered by members of the
element (ΣmemberBases). We have rewritten this section of Additional file 1: Supporting notes
to describe the calculation of element weights more clearly.

4. In Greedy Path section, in the statement "Assembly of ...... or the same path is generated
twice." Please authors explain why the same path can be generated twice.
We thank the Reviewer for bringing this detail to our attention, as it helped us clarify an issue
that was impacting a small number of loci during assembly. Based on how the method is
described, it should not be possible for the same path to be generated twice. However, there are
rare cases where a path may extend beyond the only valid end. Instead of discarding these
incomplete transcripts, they can be “trimmed back” to the nearest end in a post-processing step.
This trimmed path may be identical to a path that was already found, even if the untrimmed path
was not identical. When removing assigned weights from the graph, the trimmed path was
inadvertently being used in place of the untrimmed path, resulting in the appearance of paths
being generated twice. By correcting this bug, Bookend now avoids early termination and is
more sensitive. We re-processed all floral bud long-read and hybrid assemblies with the
updated software, and the Bookend Floral Bud annotation could identify more
reference-matching transcripts with the same level of concordance as before. We have updated
Figure 3D-E, Additional file 1: Table S3 and Additional file 2: Datasets 1,2 using Bookend v1.0.1.

5. Although the method seems to be interesting, the computational complexity is higher. The
authors should compare Bookend's running time to the previous of same kind such as [1] and
[2] mentioned above.
We agree with both Reviewers that performance of software is an important consideration. We
have looked more closely at the computation resources required for different transcript
assemblers, including total CPU time and peak memory usage. Because TransComb can only
be run on paired-end reads, we included it in the mouse single-cell analysis accompanying
Figure 4. On these samples the running time of Bookend is indeed comparable to TransComb,
and Bookend’s peak memory usage is lower than all methods tested except StringTie2.
We added Additional file 1: Fig. S4B-C to the revised manuscript to address runtime and peak
memory usage. Bookend ranked third out of five assemblers in CPU time and had the
second-lowest memory footprint.

6. In main text, are there typos in the expressions μg, ng and ug?
Thank you for pointing out our typos. In the revised manuscript, we have replaced two instances
of “ug” with “μg” in the Methods section, and one instance in the legend for Additional file 1:
Table S2.

[1] TransComb: genome-guided transcriptome assembly via combing junctions in splicing
graphs, Genome Biology (2016)
[2] TransLiG: a de novo transcriptome assembler that uses line graph iteration, Genome Biology
(2019)

Reviewer #2: The manuscript entitled "Precise Transcript Reconstruction with End-Guided
Assembly" describes a new genome-guided transcript assembler called Bookend. Although
there are already a number of RNA-Seq assembly methods, Bookend's value is in using
transcript start and end information which is embedded in the read data for more precise
transcript assembly. Its other novelty is the ability to work with RNA sequencing data from many
different platforms: short, long and end capture.
I found the manuscript very well prepared; it was written well with appropriate validation and
figures. The first author has done an outstanding job in describing the software in
supplementary material and the online user guide. I found the software to be very user friendly.
This is often overlooked and I was pleased to see the effort that was put into this aspect of the
work. Using the transcript end information is a nice idea and it appears to provide more refined
annotation, which is valuable. However transcript end information is only available from
particular sequencing protocols such as Smart-seq and PacBio, which is a limitation. My major
comments center on clarifying this limitation in more detail in the text of the manuscript.
Major comments:
1) Although the dependence on end information is discussed throughout the manuscript, I felt
that this needed to be stated upfront and more clearly. Specifically: i) In the introduction and
abstract: "with a focus on identifying and utilizing RNA 5' and 3' ends." and "utilizing RNA 5' and
3' end information contained in RNA-seq dataset", it should be clarified that end information
refers to the oligo sequences in the read data, or this could be misunderstood (e.g. to mean sets
of heuristics based on things like coverage drops) ii) the introduction could be improved by
describing which sequencing technologies do and don't provide end information. iii) In the
results: stating explicitly that Bookend labels the reads by looking for particular oligo sequences
which are platform dependent. Also saying that bookend can be run without end labels if
desired. In this case how does the algorithm work without the S and E nodes in Fig. 1?
We agree with the Reviewer that the platform-dependent nature of “RNA end information” is an
important aspect of Bookend and should be clearly communicated in the text. Indeed, only a
subset of RNA-seq methods will contain oligos informative of the 5’ and/or 3’ ends, and it would
be valuable for readers to better understand what makes a method compatible with
end-labeling. To our knowledge, 5’ labels are present only in RNA-seq libraries that make use of
a template switching oligo (TSO) during reverse transcription as we describe throughout the
text. Poly(A) tails could be labeled in most RNA-seq protocols that use an oligo-dT primer during
reverse transcription. We have attempted to clarify what we refer to as end information and that
only a subset of protocols generate RNA end information in the Abstract and Introduction of therevised text. We also describe which technologies produce end information throughout the
Results. Moreover, we added a paragraph to the Discussion explicitly stating this limitation to
the Bookend approach, as well as recommendations for users to generate new or incorporate
existing RNA end information to improve transcript assembly.
If Bookend is run without requiring end labels (bookend assemble –allow_incomplete or
bookend condense), then the deadEndPenalty in the Algorithm ExtensionScore is always set to
1. This makes it possible to output paths that fail to join the S and E nodes, and each path
simply extends until no more extensions can be made. The effect of this option on the behavior
of the ExtensionScore algorithm is described in the subsection “Greedy Paths” of Additional file
2: Supporting notes. We provide an example of Bookend run in this way on single-cell data in
Additional file 1: Fig. S4C-D of the revised manuscript.

2) The limitations of the methods should be described. For example, without end labeled data
(which is the majority of RNA sequencing data generated), it only performs with similar accuracy
to existing methods like Stringtie (Fig. 2D-F). Is there a way this could be overcome. For
example, is it worth a users seeking public Smart-seq or PacBio data to compliment their other
datasets for use with Bookend? Even if it's from a different tissue?
As we showed, Bookend does not strongly outperform standard assemblers in any category if
end-labeled reads are unavailable. Unfortunately, accurate full-length transcript annotation may
not be possible without end data, so we would strongly recommend to users that their datasets
be supplemented with tissue-matched Smart-seq or long-read libraries. If this is infeasible, a
number of public datasets are available, including a very large collection of CAGE libraries from
the FANTOM consortium on many tissues and cell types in human and mouse (Kawaji et al.
2017 Scientific Data). Additionally, the Tabula Projects organized by the Chan Zuckerberg
Biohub are rapidly producing comprehensive single-cell atlases of end-labeled RNA-seq from
the mouse, fly, human and others. In the revised manuscript, we suggest that users integrate or
generate datasets containing end-labeled reads to increase the performance of transcript
annotation.
Even with data that can be end-labeled, genes with low expression are unlikely to be assembled
due to a lack of end reads (Fig. 2A/2C). This may explain why other assemblers produce more
novel transcripts. Although the authors compare to reference annotations, these are known to
be incomplete and in particular RefSeq is known to miss many transcripts reported by other
annotations. Similarly, PacBio data is generally low coverage, so rare transcripts will be missed.
Often the aim of transcript assembly is to improve differential expression and it is not clear how
Bookends performs in this regard. Can the authors comment on this? They could also state the
fraction of reads mapping to transcripts for each of the different assemblers, which is an
important alternative metric to consider.
We thank the Reviewer for suggestions on alternative ways to evaluate transcript annotation
quality. We used various external and internally produced datasets to assess the quality of
transcript annotations produced by Bookend. For example, in the mESC assemblies wecompared non-RefSeq transcript models to mESC CAGE, 3P-seq, and Capture Long-Seq
(CLS) datasets to validate models missed by the reference (Fig. 4). Novel intergenic transcripts
are one of the largest classes of transcripts produced by other assemblers. Bookend assembled
97% fewer novel intergenic transcripts than StringTie2; despite this, CLS data validated an
average of 16 intergenic Bookend transcripts per cell, but only validated an average of 3
intergenic StringTie2 transcripts per cell. For this reason, we strongly believe that the
overwhelming majority of novel transcripts produced by other assembly methods are technical
artifacts.
Conceptually we agree that a higher quality annotation should enable the user to make more
biologically meaningful inferences from their data. To determine whether a transcript assembly
produced by Bookend is sensitive enough to give insight into tissue-specific gene expression,
we quantified a collection of previously published low-input 5’ end libraries from dissected floral
tissues. We found that the set of transcription start sites described in the Bookend Floral Bud
assembly can explain a higher percentage of reads from every tissue type than the existing
reference annotations (i.e. TAIR10 and Araport11). Moreover, transcript models newly reported
by Bookend are overrepresented in the set of tissue-enriched genes including interesting
examples in male reproductive organs (i.e. anthers). We have expanded Figure 3 with panels F
through L to discuss our differential gene expression analysis, and highlight how Bookend
enables new insights into tissue-specific transcript isoforms in plant reproductive organs.

3) The computational resources required to run Bookend should be compared against
alternative assemblies. Bookend does not need to be the fastest, but it should be shown that
this is not a limitation to the software's use in practice.
We thank both Reviewers for recommending a comparison of computational resources. As
written in response to Reviewer #1’s comment above, we added Additional file 1: Fig. S4B-C to
the revised manuscript to address runtime and peak memory usage. Bookend ranked third out
of five assemblers in CPU time and had the second-lowest memory footprint.

Minor comments/questions:
1) Figure 1C - Why don't the nodes corresponding to single frag elements (e.g. 4) have
incoming edges in the directed graph (e.g. 3->4 rather than 4->3)?
Your question highlights an important detail of the Overlap Graph defined by Bookend. In Figure
1C, a directed edge can only exist from element 1 to element 2 if 2 extends 1, i.e. if 2 shares at
least one overlapping member with 1 and at least one member of a higher index. Because
element 4 only has one member (i.e. frag d), it cannot extend. Instead, element 4 is contained
by four other elements (1, 3, 5, 6), so its weight will be redistributed proportionally to these four
elements by the subroutine Resolve Containment prior to assembly. Because single-frag
elements can never be traversed by a path they are shown in gray in Figure 1C. Single-frag
elements contain no unique structural information and their weight is accounted for by the
elements that contain them. We have clarified our definition of "extend" in the Methodssubsection Assembly Algorithms (Calculate Overlap Matrix) of the revised manuscript, and a
fuller description is provided in the “Calculate Overlap Matrix” section of Additional file 1:
Supporting notes.

2) Figure 3E - If Bookend is already established as the best assembler in 3D, why not show the
difference of the Bookend hybrid compared to Bookend non-hybrid? Also, is the Stringtie
assembly hybrid here?
In Figure 3D it is important to note that the top two methods (Iso-seq3, ToFU) do not assemble
long reads; these two techniques cluster and filter the transcript models described by long
reads, but they do not attempt to synthesize transcript models that aren’t explicitly present in the
input. To clarify this distinction we have changed the title of Figure 3D to “PacBio long-read
processing tools”. Figure 3E was an attempt to address whether using assembly algorithms on
long reads gives more desirable results than a simpler method like clustering. For this reason
we compared the possible methods of assembly against the best-performing method that is not
based on assembly.
The previous version of StringTie2 did not have the option to perform hybrid assembly with short
and long reads. However, while our manuscript was under review the authors of StringTie2
uploaded a manuscript to bioRxiv describing how they implemented hybrid assembly (Shumate
et al. 2021). We have updated Figure 3E and Additional file 1: Table S3 of the revised
manuscript to include the performance of hybrid assembly with StringTie2.

3) The explanation for read-through transcripts being removed by Bookends is the addition of
end information in the assembly, but read-through transcripts should similarly have starts and
ends as they are genuine transcripts. However read-through events are rare, so perhaps these
are filtered out because the coverage of end-labeled reads is low?
The Reviewer is correct that read-through transcripts can be genuine events, but are usually
rare and incompletely processed transcripts in wild-type tissues. These transcripts, unless
particularly abundant, are generally unwanted in an annotation. For this reason we apply a
coverage-based filter against putative read-through transcripts that requires the read-through to
have higher estimated coverage than at least one of the two genes it contains.
We believe that nearly all putative read-through transcripts reported by non-end-guided
assemblers in our datasets are assembly artifacts. Additional file 1: Table S2 shows that all
methods that do not incorporate end information generate over 1000 assemblies that are
“fusions” of two adjacent genes. However, our PacBio libraries validated only 6% or fewer of
these fused assemblies as genuine transcripts spanning two genes (Fig. 2G).

4) The final line in "Discussion", "Bookend enables the wholesale reannotation of genomes at
single-cell resolution...". I think this should be reworded. Annotations are usually considered
universal (ie. non-tissue or cell specific). I think the authors are referring here to annotating the
expressed transcripts at single-cell level resolution.

We think that genome annotations should contain the full set of transcripts including those that
are tissue or cell specific. We have changed the wording of the final sentence in the revised
manuscript to clarify Bookend’s capabilities in this regard.
